# SALL4 is a CRL3^REN/KCTD11^ substrate that drives Sonic Hedgehog-dependent medulloblastoma

**DOI:** 10.1038/s41418-023-01246-6

**Published:** 2023-12-07

**Authors:** Ludovica Lospinoso Severini, Elena Loricchio, Shirin Navacci, Irene Basili, Romina Alfonsi, Flavia Bernardi, Marta Moretti, Marilisa Conenna, Antonino Cucinotta, Sonia Coni, Marialaura Petroni, Enrico De Smaele, Giuseppe Giannini, Marella Maroder, Gianluca Canettieri, Angela Mastronuzzi, Daniele Guardavaccaro, Olivier Ayrault, Paola Infante, Francesca Bufalieri, Lucia Di Marcotullio

**Affiliations:** 1 Department of Molecular Medicine, University of Rome La Sapienza, 00161 Rome, Italy; 2 Institut Curie, PSL Research University, CNRS UMR, INSERM, 91401 Orsay, France; 3 Centro Nazionale per il Controllo e la Valutazione dei Farmaci, Istituto Superiore di Sanità, 00161 Rome, Italy; 4 Université Paris Sud, Université Paris-Saclay, CNRS UMR, INSERM U, 91401 Orsay, France; 5 Department of Experimental Medicine, University of Rome La Sapienza, 00161 Rome, Italy; 6 Istituto Pasteur-Fondazione Cenci Bolognetti, University of Rome La Sapienza, 00161 Rome, Italy; 7 Department of Pediatric Haematology and Oncology, and Cell and Gene Therapy, Bambino Gesù Children’s Hospital, IRCCS, 00165 Rome, Italy; 8 Department of Biotechnology, University of Verona, 37134 Verona, Italy

**Keywords:** CNS cancer, Preclinical research

## Abstract

The Sonic Hedgehog (SHH) pathway is crucial regulator of embryonic development and stemness. Its alteration leads to medulloblastoma (MB), the most common malignant pediatric brain tumor. The SHH-MB subgroup is the best genetically characterized, however the molecular mechanisms responsible for its pathogenesis are not fully understood and therapeutic benefits are still limited. Here, we show that the pro-oncogenic stemness regulator Spalt-like transcriptional factor 4 (SALL4) is re-expressed in mouse SHH-MB models, and its high levels correlate with worse overall survival in SHH-MB patients. Proteomic analysis revealed that SALL4 interacts with REN/KCTD11 (here REN), a substrate receptor subunit of the Cullin3-RING ubiquitin ligase complex (CRL3^REN^) and a tumor suppressor lost in ~30% of human SHH-MBs. We demonstrate that CRL3^REN^ induces polyubiquitylation and degradation of wild type SALL4, but not of a SALL4 mutant lacking zinc finger cluster 1 domain (ΔZFC1). Interestingly, SALL4 binds GLI1 and cooperates with HDAC1 to potentiate GLI1 deacetylation and transcriptional activity. Notably, inhibition of SALL4 suppresses SHH-MB growth both in murine and patient-derived xenograft models. Our findings identify SALL4 as a CRL3^REN^ substrate and a promising therapeutic target in SHH-dependent cancers.

## Introduction

Deregulations in developmental signaling pathways are crucial events in the pathogenesis of cancer and deciphering the complex networks that govern their activity is fundamental to design novel and effective therapeutic options. Sonic Hedgehog (SHH) signaling is a major developmental pathway, highly conserved during evolution and orchestrated at multiple levels. In the cerebellum, SHH stimulates the proliferation of granule neuron precursors (GNPs) and the aberrant activation of the SHH signaling in these cells is responsible for medulloblastoma (MB) onset, the most common malignant pediatric brain tumor [1]. In the last decade, whole genome sequencing approaches have defined specific mutational spectra and epigenetic profiling leading to the identification of distinct subgroups, namely Wingless (WNT), Sonic Hedgehog (SHH), and non-WNT/non-SHH, comprising Group 3 (G3) and Group 4 (G4) [2]. Among them, SHH-MB is the most genetically understood further classified in four molecular subtypes (SHH-MB alpha, beta, gamma, delta) [3] and represents ~30% of all MBs [1]. Mutations in key components of the SHH pathway and cytogenetic alterations lay the pathogenetic foundation for this subgroup [4].

A growing body of evidence has underlined the existence of an intricate network of molecular mechanisms that control SHH signaling. However, how alterations of such events are involved in SHH-MB remains unclear. The signaling is triggered following the interaction of the SHH ligand with its receptor Patched (PTCH), thus relieving the repression on the co-receptor Smoothened (SMO) and leading to the activation of the GLI transcription factors (GLI1, GLI2, GLI3). GLI1 is the main downstream effector of the pathway that, by driving its own expression, provides a positive feedback loop and reinforces the signaling strength [5]. Dissecting the molecular circuitry that controls GLI1 activity is needed to unveil the mechanisms responsible for SHH-driven diseases.

Ubiquitylation and acetylation are post-translational modifications that finely regulate GLI1 activity. Previously we reported that GLI1-mediated transcription is controlled by a multiprotein complex including HDAC1, a deacetylase upregulated in SHH-MB [6, 7], and REN/KCTD11 (here REN), the substrate-receptor subunit of the CRL3^REN^ ubiquitin ligase, encoded by a gene localized on chromosome 17p and frequently deleted in SHH-MB [6, 8]. REN belongs to the “KCTD containing Cullin3 adaptor suppressor of Hedgehog” (KCASH) protein family [9]. It is involved in neuronal progenitor development [10] and acts as an antagonist of the SHH pathway by inducing ubiquitylation and degradation of HDAC1, which in turn deacetylates GLI1 promoting its activation [6]. This acetylation/ubiquitylation interplay functions as a key transcriptional checkpoint of SHH signaling that deserves further investigations.

By affinity purification coupled to mass spectrometry, we identified the Spalt-like transcriptional factor 4 (SALL4) as a binding partner and substrate of CRL3^REN^, which induces the polyubiquitylation and proteasome-mediated degradation of SALL4. SALL4 is a zinc finger transcription factor belonging to *spalt* (*sal*) gene family, highly expressed in embryonic stem cells (ESCs) and crucial for the maintenance of pluripotency [11–14]. SALL4 protein presents a conserved motif of 12 amino acids at the N-terminal that interacts with the nucleosome remodeling and histone deacetylase (NuRD) complex [15, 16], and seven zinc finger (ZF) motifs organized in three clusters (ZFC1, ZFC2, ZFC4) with an additional single ZF near the N-terminal [17]. SALL4 is downregulated in most adult tissues, but it is re-expressed in various human malignancies [18–24]. It acts as a transcriptional repressor when associated with NuRD complex by inhibiting the expression of pro-apoptotic and tumor suppressor genes [25, 26].

Here, we show a novel mechanism of action of SALL4 in SHH-MB pathogenesis whereby its re-expression, due to loss of REN, drives the activation of GLI1. Specifically, we found that SALL4 forms a trimeric complex with GLI1 and HDAC1 to induce GLI1 protein deacetylation, thus enhancing its activity. Remarkably, inhibition of SALL4 arrests tumor growth in vitro and in vivo. These findings highlight SALL4 as a crucial player of the SHH pathway and innovative target for tailored SHH-MB therapies.

## Results

### The CRL3^REN^ complex binds SALL4 and induces its proteasome-mediated degradation

**Fig. 1:**
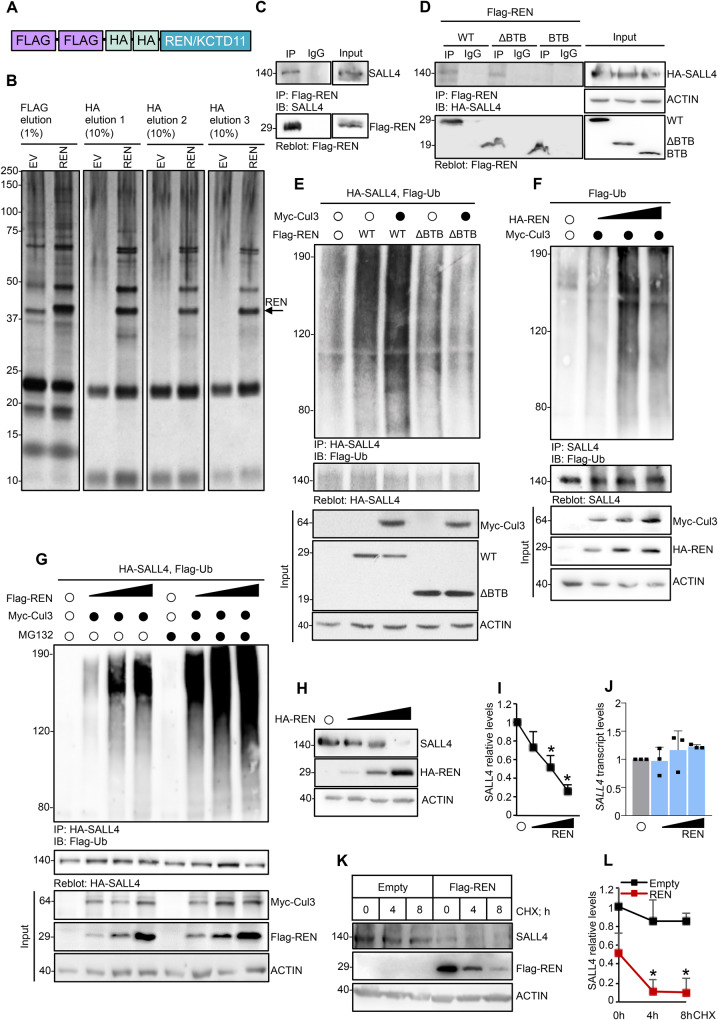
REN binds and ubiquitylates SALL4 promoting its proteasome-mediated degradation. **A** HEK293T cells were transfected with plasmids encoding for Flag-HA epitope tagged REN/KCTD11 or an empty vector (EV). **B** Cells were treated with the proteasome inhibitor MG132 and lysed. Whole cell extracts were immunoprecipitated with anti-Flag resin and eluted with Flag peptide. A second immunoprecipitation was carried out with an anti-HA antibody, which was followed by three sequential elutions with 1% SDS, as indicated. Immunocomplexes were resolved by SDS-PAGE. The gel was then stained by silver stain for protein visualization. Co-IPs of **C** Flag-REN and endogenous SALL4, or **D** Flag-REN WT or its mutants (ΔBTB, BTB) and exogenous SALL4 from HEK293Ts transiently transfected with indicated plasmids. IPs of **E** HA-SALL4 or **F** endogenous SALL4 from HEK293Ts transiently transfected with indicated plasmids. **G** IP of HA-SALL4 from HEK293Ts transiently transfected with indicated plasmids and treated with MG132 (50 μM, 4 h) to block proteasome, or DMSO as control. In E, F, and G anti-Flag antibody was used to detect the SALL4 polyubiquitylated forms; anti-HA or anti-SALL4 antibodies were used to re-probe blots to assess the levels of immunoprecipitated protein. Total protein lysates are shown in the Input. **H**, **K** Representative immunoblotting and **I**, **L** densitometric analysis of SALL4 protein levels in Med1-MB cells transiently transfected with indicated plasmids, and treated in K with CHX (100 μg/mL) up to 8 h to block protein synthesis. **J** qRT-PCR analysis of *SALL4* expression in Med1-MB cells overexpressing increasing amounts of HA-REN. Representative immunoblotting of *n* = 3 biological replicas with similar results are shown in C–H, and K. Densitometric analysis in I and L, normalized on endogenous actin, represent the mean of *n* = 3 independent experiments ± SD. Data in J are normalized to endogenous *Gapdh* and *Hprt* control, expressed as the fold change (FC) versus the control sample value, and represent the mean of *n* = 3 independent experiments ± SD. *p < 0.05 calculated with two-sided Student’s t-test.

To identify novel REN binding partners involved in SHH signaling and tumorigenesis, we performed immunoaffinity-purification of Flag-HA-tagged wild type (WT)-REN (Fig. 1A) ectopically expressed in HEK293T cells, followed by mass spectrometry analysis. In addition to proteins known to directly bind REN, such as Cul3, RBX1, and KCTD15 (Supplementary Fig. 2A) [6, 27], we recovered various potential REN interactors (Fig. 1B) including SALL4, a cell stemness regulator aberrantly activated in several types of human cancers [18–24].

Interestingly, using the expression profile from primary SHH-MB samples and the associated clinical information (Cavalli dataset27; accession number: GSE85217; visualized using R2 platform, https://r2.amc.nl), we observed that high SALL4 expression is related to worse prognosis in SHH-MB patients (Supplementary Fig. S1A), specifically in alpha, beta, and delta but not in gamma SHH-MB subtype which expresses lower SALL4 levels compared to the others (Supplementary Fig. S1B–D). This evidence prompted us to pursue our investigation on SALL4 as REN interactor.

We first confirmed the interaction between REN and SALL4. Co-immunoprecipitation experiments demonstrate that REN binds both exogenous and endogenous SALL4 in HEK293T cells (Supplementary Fig. S2B and Fig. 1C, respectively). Then, we investigated the region of REN responsible for its interaction with SALL4. REN contains an N-terminal Broad-Complex, Tramtrack and Bric a brac (BTB) domain that is known to mediate the recruitment of Cul3 [28]. We transfected HEK293Ts with HA-SALL4 together with WT-, ΔBTB- (a BTB domain-deleted mutant) or BTB- (a mutant containing only BTB domain) REN. Whereas WT- and ΔBTB-REN interact with SALL4, the BTB mutant does not indicating that the BTB domain is not required for the interaction of REN with SALL4 (Fig. 1D).

To assess if SALL4 is a substrate of the CRL3^REN^ ubiquitin ligase complex, we examined the ability of REN to promote SALL4 ubiquitylation in cultured cells. Overexpression of REN promotes the polyubiquitylation of SALL4 that is further increased by Cul3, whereas the ΔBTB-REN mutant is ineffective (Fig. 1E). Increasing amounts of REN result in a progressive increment in the polyubiquitylation of both exogenous and endogenous SALL4 (Supplementary Fig. S2C and Fig. 1F, respectively), and a significant accumulation of polyubiquitylated SALL4 is further observed following treatment with the proteasome inhibitor MG132 (Fig. 1G). Accordingly, SALL4 protein stability progressively decreases in the presence of increasing amounts of REN in Med1-MB cell lines, derived from a *Ptch1*^+/−^;lacZ mouse model which spontaneously develops MB due to heterozygous deletion of *Ptc* gene [29, 30] (Fig. 1H, I). Remarkably, *SALL4* transcript levels are not modulated (Fig. 1J). Moreover, cycloheximide (CHX) assay shows that REN impinges upon the half-life of SALL4 (Fig. 1K, L). These results demonstrate that the CRL3^REN^ ubiquitin ligase complex specifically targets SALL4 for polyubiquitylation and proteasomal degradation.

**Fig. 2:**
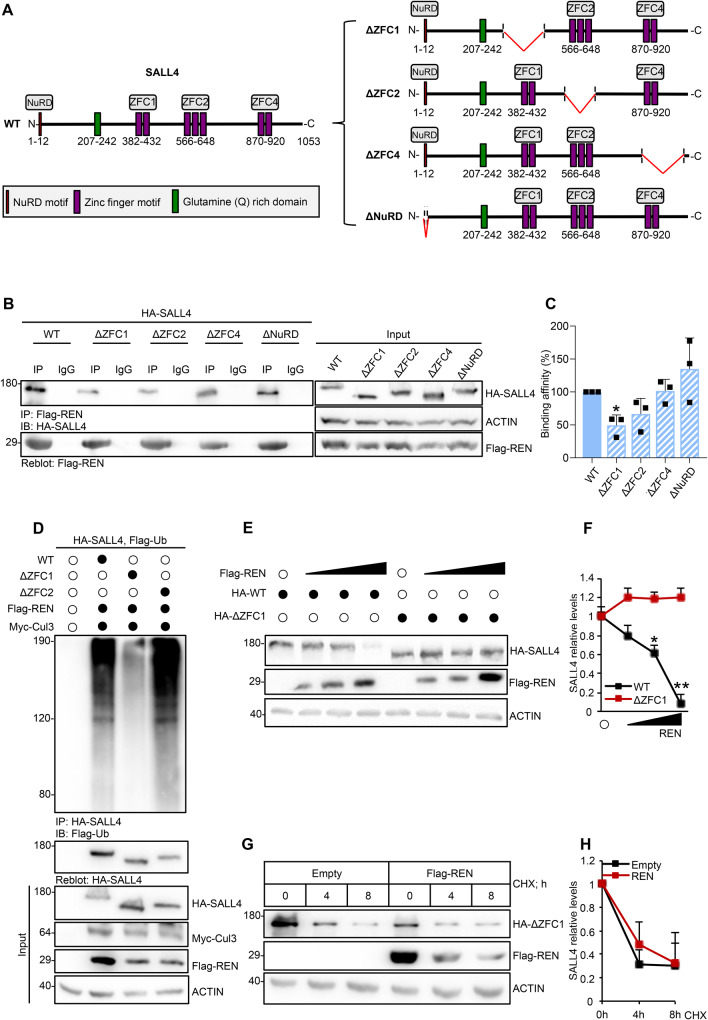
ΔZFC1-SALL4 is a degradation-defective mutant. **A** Schematic representation of SALL4 WT (left panel) and SALL4 mutants (right panel): ΔZFC1-SALL4 (Δ320-486 aa); ΔZFC2-SALL4 (Δ551-662 aa); ΔZFC4-SALL4 (Δ859–1028 aa); ΔNuRD-SALL4 (Δ1-12 aa). **B** Co-IPs of Flag-REN and ectopic SALL4 (WT or mutants) from HEK293Ts transiently transfected with indicated plasmids and **C** relative binding affinities (%) normalized to immunoprecipitated Flag-REN (mean of *n* = 3 individual experiments ± SD). **D** IP of HA-SALL4 from HEK293Ts transiently transfected with indicated plasmids. WT- and ΔZFC2-SALL4 are used as controls. Anti-Flag antibody was used to detect the SALL4 polyubiquitylated forms; anti-HA antibody was used to re-probe blot to assess the levels of immunoprecipitated protein. Total protein lysates are shown in the Input. **E** Protein levels of WT- and ΔZFC1-SALL4 in HEK293Ts transfected with a vector encoding REN and **F** relative densitometric analysis. **G** ΔZFC1-SALL4 protein stability in HEK293Ts transfected with the indicated plasmids and treated with CHX (100 µg/mL) at different time points, with **H** relative densitometric analysis. Representative immunoblotting of *n* = 3 biological replicas with similar results are shown in B, D, E, and G. Actin-normalized densitometric analysis in F and H represent the mean of *n* = 3 independent experiments ± SD; *p < 0.05; **p < 0.01 calculated with two-sided Student’s *t*-test.

To identify the region of SALL4 ubiquitylated by CRL3^REN^, we analysed a set of SALL4 mutants lacking NuRD (ΔNuRD) [15, 16] or clusters of ZF motifs (ΔZFC1, ΔZFC2, and ΔZFC4, involved in the binding to DNA and in protein-protein interaction [12, 17, 31–33]) (Fig. 2A). The ΔZFC1-SALL4 mutant shows a significant reduction in the binding affinity for REN compared to the WT-SALL4 and the ΔNuRD-, ΔZFC2-, ΔZFC4-SALL4 mutants (Fig. 2B, C) and it is insensitive to the CRL3^REN^–mediated polyubiquitylation (Fig. 2D). Accordingly, neither protein abundance of ΔZFC1-SALL4 (Fig. 2E, F) nor its degradation rate (Fig. 2G, H) are modulated by REN.

### SALL4 positively controls the SHH signaling pathway

**Fig. 3:**
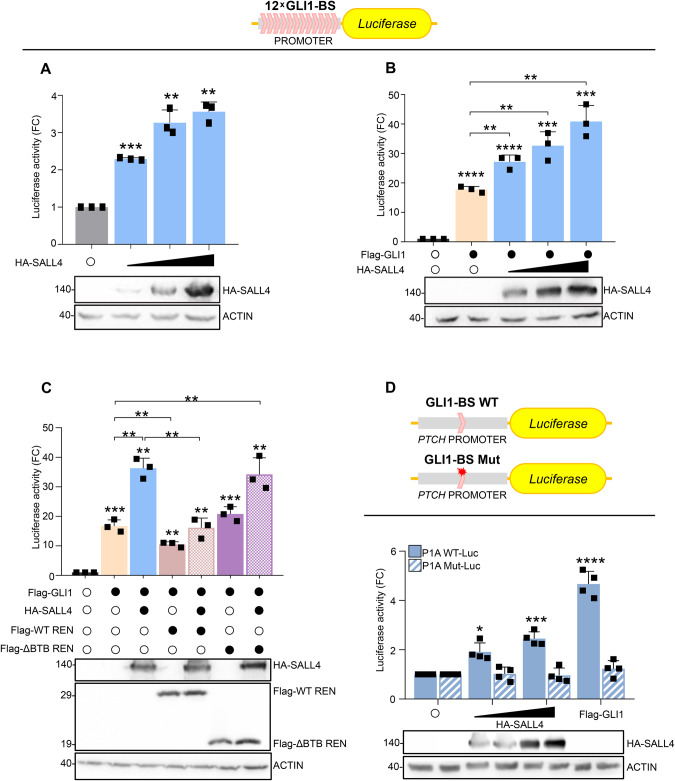
SALL4 positively regulates the SHH pathway. The transcriptional activity of **A** endogenous and **B**. **C** exogenous GLI1 has been assessed in the presence of ectopic SALL4 in HEK293Ts transiently transfected with a *Firefly* luciferase reporter gene under the control of a synthetic promoter containing 12 binding sites for GLI1 (12 × GLI1-BS), pRL-TK *Renilla* as normalization control, and the indicated plasmids. **D** GLI1-mediated transcription has been evaluated in HEK293Ts transiently transfected with constructs expressing *Firefly* luciferase under the control of *PTCH* promoter presenting a conserved (P1A WT-Luc) or mutated (P1A Mut-Luc) GLI1-BS, pRL-TK *Renilla* as normalization control, and the indicated plasmids. Luciferase analyses were performed 24 h after transfection. Data in A–C represent the mean of *n* = 3 independent experiments ± SD; data in D represent the mean of *n* = 4 independent experiments ± SD. All data are expressed as FC versus empty vector. Representative immunoblotting of *n* = 3 biological replicas with similar results showing the expression levels of transfected plasmids are reported. *p < 0.05; **p < 0.01; ***p < 0.001; ****p < 0.0001 calculated with two-sided Student’s t-test.

The role of REN as negative regulator of the SHH pathway [6] prompted us to wonder whether SALL4 is functionally connected to this crucial signaling. SALL4 expression enhances the transcription of a GLI1-responsive luciferase reporter [34] in a dose-dependent manner (Fig. 3A, B) and this effect is counteracted by WT-REN, but not by its ΔBTB mutant (Fig. 3C). Interestingly, SALL4 induces the transcription of a *PTCH*-dependent luciferase reporter having a conserved GLI1 binding site (GLI1-BS) in its promoter (P1A WT-Luc), while it is not effective when GLI1-BS is mutated (P1A Mut-Luc) [35] (Fig. 3D). Given that we did not find SALL4 binding sites on *GLI1* [36], our findings indicate that the SALL4-mediated regulation of the SHH pathway requires the integrity of GLI1 consensus element.

### SALL4 enhances SHH signaling activity through HDAC1-mediated deacetylation of GLI1

**Fig. 4:**
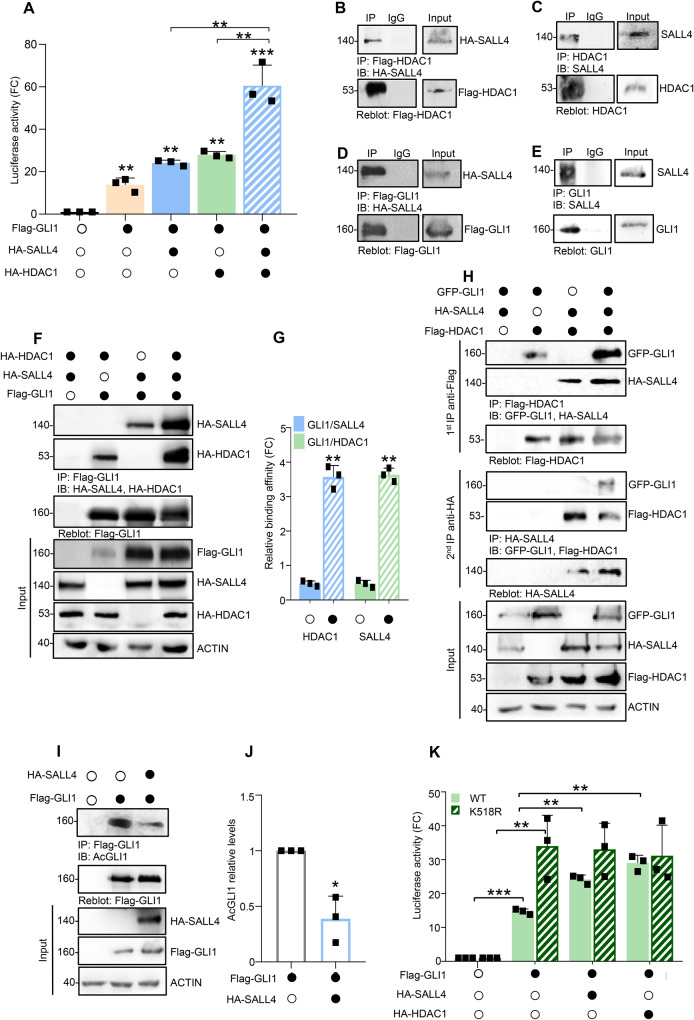
SALL4 cooperates with HDAC1 in reducing GLI1 acetylation levels. **A** GLI1-induced transcription has been assessed in HEK293Ts transiently transfected with 12 × GLI1-BS *Firefly* luciferase, pRL-TK *Renilla* as normalization control, and the indicated plasmids; data are expressed as FC versus empty vector. Co-IPs of SALL4 and ectopic or endogenous HDAC1 (**B**, **C**, respectively), or ectopic or endogenous GLI1 (**D**, **E**, respectively) in HEK293Ts transiently transfected as indicated. **F** Co-IPs of GLI1, SALL4, and HDAC1 in HEK293Ts transiently transfected with vectors expressing the indicated plasmids. **G** Densitometric analysis of relative GLI1/SALL4 and GLI1/HDAC1 binding affinities, expressed as FC versus relative controls, are normalized to immunoprecipitated Flag-GLI1. **H** HEK293Ts were transfected with different combinations of indicated plasmids. Protein lysates were immunoprecipitated with anti-Flag agarose beads. One-third of immunocomplexes was probed with antibodies to the indicated proteins (1^st^ IP), whereas two-thirds were subjected to two elutions with Flag-peptide and re-immunoprecipitated with HA-agarose beads followed by immunoblotting as indicated (2^nd^ IP). **I** Co-IP of Flag-GLI1 in HEK293Ts transiently transfected with indicated plasmids. The acetylation levels of GLI1 protein at K518 residue have been assessed by using an in-house generated antibody [39]. Total protein lysates are shown in the Input. **J** Densitometric analysis of relative AcGLI1 levels, normalized to immunoprecipitated Flag-GLI1. **K** GLI1-dependent transcription has been assessed in HEK293Ts transiently transfected with 12 × GLI1-BS *Firefly* luciferase, pRL-TK *Renilla* as normalization control, and indicated plasmids. Data are expressed as FC versus empty vector. Representative immunoblotting of *n* = 3 biological replicas with similar results are shown in B–F, H, and I. Densitometric analysis in G and J represent the mean of *n* = 3 independent experiments ± SD; *p < 0.05 *p < 0.05; **p < 0.01; ***p < 0.001 calculated with two-sided Student’s t-test.

REN prevents the HDAC1-mediated deacetylation of GLI1 protein, a key regulatory mechanism in the control of the SHH transcriptional output [6]. Therefore, we postulated a potential cooperation between SALL4 and HDAC1 in the enhancement of SHH pathway activation. To test our hypothesis, we carried out GLI1-responsive luciferase assays demonstrating that co-expression of SALL4 and HDAC1 increases GLI1 transcriptional activity that is significantly higher than the effect induced by SALL4 and HDAC1 transfected alone (Fig. 4A). In this regard, we confirmed that SALL4 binds both exogenous and endogenous HDAC1 (Fig. 4B, C, respectively) and demonstrated, for the first time, the ability of SALL4 to interact with GLI1 (Fig. 4D, E) in SHH-MB cell lines (Med1-MB). Notably, we observed that GLI1/SALL4 and GLI1/HDAC1 binding affinities are significantly strengthened when the three proteins are co-expressed in cultured cells (Fig. 4F, G). Furthermore, we expressed epitope tagged SALL4, HDAC1, and GLI1 in HEK293Ts in different combinations [37, 38] and performed two sequential immunoprecipitations demonstrating that SALL4, HDAC1, and GLI1 are assembled in a trimeric complex (Fig. 4H).

The deacetylation of GLI1 at lysine 518 (K518) mediated by HDAC1 is critical to enhance GLI1 activity [6]. Interestingly, we observed that SALL4 decreases the levels of Acetyl-K518-GLI1 (AcGLI1) (Fig. 4I, J) detected through a specific in-house generated antibody [39]. Additionally, SALL4 overexpression is not effective in inducing the transcriptional activity of the not acetylable K518R-GLI1 mutant [6] (Fig. 4K). These results indicate that SALL4 cooperates with HDAC1 to enhance SHH signaling through the regulation of GLI1 acetylation.

### SALL4 knock-down inhibits the proliferation of SHH-MB cells by eliciting GLI1 acetylation and counteracting SHH signaling

**Fig. 5:**
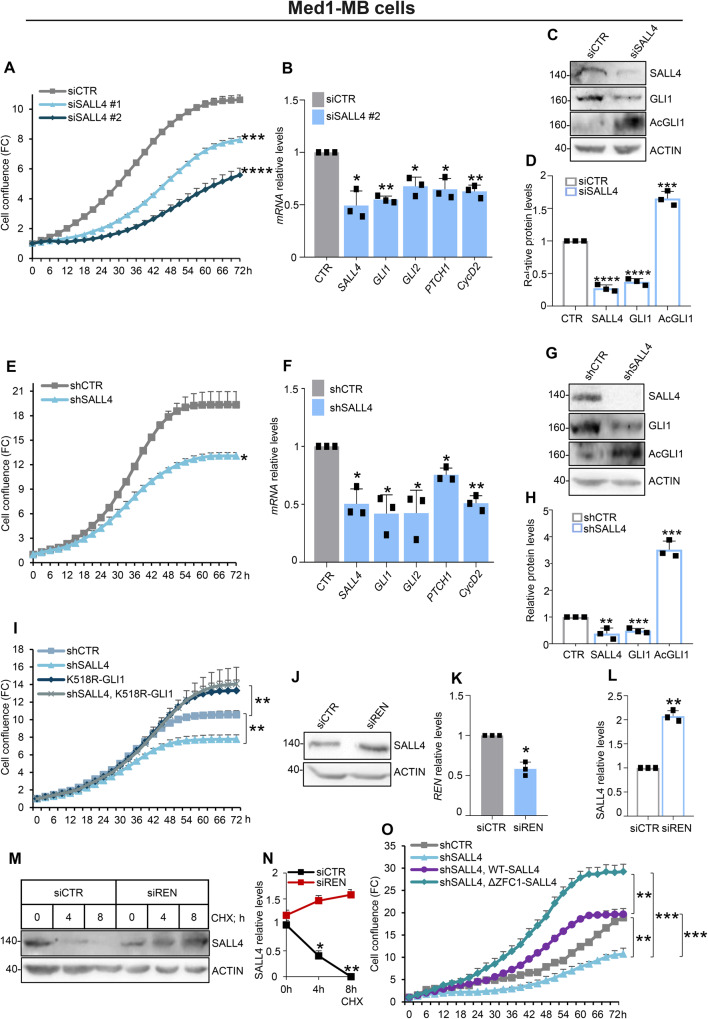
SALL4 knock-down inhibits the proliferation of SHH-MB cell lines. Med1-MB cells have been transiently transfected with (**A–D**) two small interfering RNAs (siRNAs) or (**E–H**) a short hairpin RNAs (shRNA) targeting SALL4 or a non-relevant control sequence (siCTR or shCTR). Cell proliferation in A and E has been measured as cell confluence calculated using the IncuCyte® Zoom software by phase-contrast images. **B**, **F** The transcript levels of SHH signature target genes are expressed as FC versus relative controls. **C**, **G** Protein levels of SALL4, GLI1, and AcGLI1 and **D**, **H** relative densitometric analysis are shown. **I** Med1-MB cells have been transiently transfected as indicated, and proliferation is expressed as cell confluence calculated using the IncuCyte® Zoom software by phase-contrast images. **J** SALL4 protein levels in Med1-MB cells transfected with siREN or siCTR. **K** qRT-PCR of *REN* silencing in this setting is shown, as well as **L** SALL4 densitometric analysis. **M** SALL4 half-life in Med1-MB cells transfected as indicated and then treated with CHX (100 µg/mL) up to 8 h; densitometric analysis is shown in **N**. **O** Med1-MB cells have been transiently transfected as indicated, and proliferation is expressed as cell confluence calculated using the IncuCyte® Zoom software by phase-contrast images. Data in A, E, I, and O are normalized to cell scans at time 0 and expressed as FC, and represent the mean of *n* = 3 independent experiments ± SD. Representative immunoblotting of *n* = 3 biological replicas with similar results are shown in C, G, J, and M. Actin-normalized densitometric analysis in D, H, L, and N represent the mean of *n* = 3 independent experiments ± SD. Data in B, F, and K are normalized to endogenous *Gapdh* and *Hprt* control expressed as FC respect to the control sample value and represent the mean of *n* = 3 independent experiments ± SD. *p < 0.05; **p < 0.01; ***p < 0.001; ****p < 0.0001 calculated with two-sided Student’s t-test.

Next, we investigated the role of SALL4 on SHH-dependent tumor growth. To this end, we silenced SALL4 expression in Med1-MB cells by using two different short interfering RNAs (siSALL4 #1 and #2). SALL4 knock-down results in a decrease in cell proliferation compared to control cells (Fig. 5A). Accordingly, SHH transcriptional signature is downregulated (Fig. 5B) and AcGLI1 levels are increased in SALL4-silenced cells (Fig. 5C, D). Similar results have been observed in Med1-MB cells infected with purified lentiviral particles encoding a short hairpin RNA targeting murine SALL4 (shSALL4) (Fig. 5E–H). Of note, the overexpression of the K518R-GLI1 mutant rescues the defective proliferation of Med1-MB cells upon SALL4 depletion (Fig. 5N). These findings indicate that SALL4 sustains SHH-MB cells proliferation favouring GLI1 deacetylation and the SHH pathway activation.

Given that SALL4 is a substrate of the CRL3^REN^ complex, silencing of REN increases SALL4 protein abundance and its half-life in Med1-MB cells (Fig. 5J–N). In agreement with our previous data (Fig. 2), the ΔZFC1-SALL4 mutant rescues the growth of SALL4-silenced cells (Fig. 5O). Overall, these results demonstrate that the REN-mediated degradation of SALL4 is a crucial event for the proliferation of SHH-MB cells, and that alterations of this mechanism could favour SHH-MB tumorigenesis.

### SALL4 genetic depletion inhibits SHH-MB cells growth in vitro

**Fig. 6:**
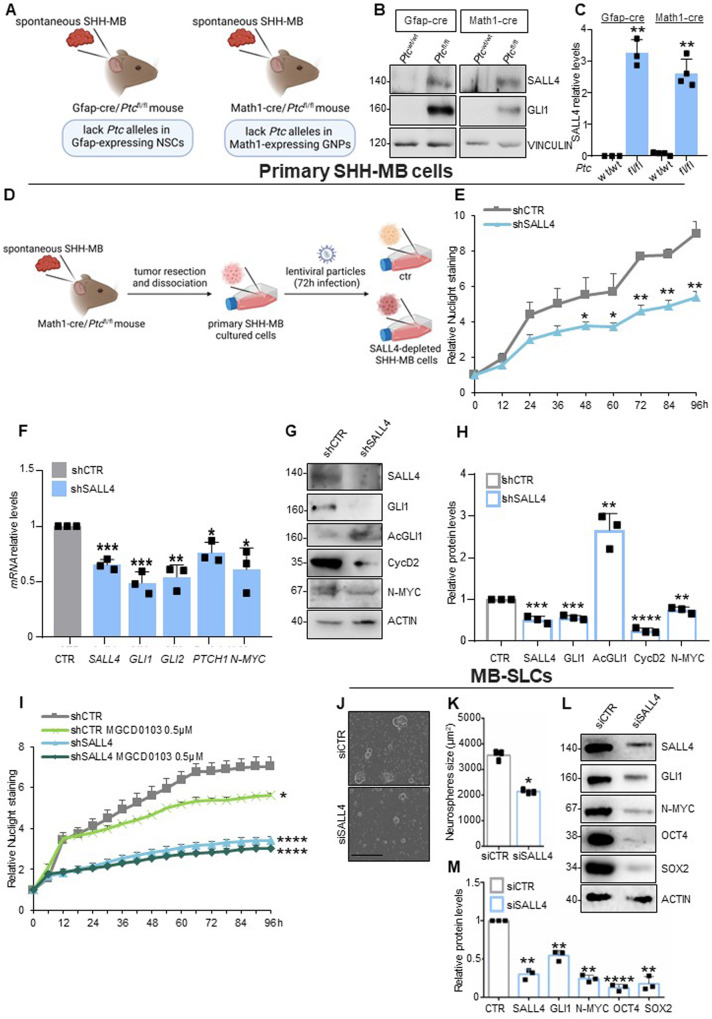
SALL4 inhibition impairs SHH-dependent tumor cell growth in vitro. **A-C** SALL4 levels have been evaluated in protein lysates of SHH-MB tissues from Gfap-cre/*Ptc*^fl/fl^ and Math1-cre/*Ptc*^fl/fl^ mice and compared to the cerebella of healthy siblings (*Ptc*^wt/wt^). **D** Primary SHH-MB cells have been infected with lentiviral particles expressing shSALL4 or shCTR. **E** Cell proliferation has been measured as Nuclight relative staining calculated at indicated time points by using the IncuCyte® Zoom software. **F** Transcript levels of SHH target genes and **G** expression of SHH-related proteins are shown, with **H** relative densitometric analysis. **I** SALL4-depleted primary SHH-MB cells, and control cells, have been treated with MGCD0103 (0.5 μM, 24 h) or DMSO as control. Cell proliferation has been measured as relative Nuclight staining calculated using IncuCyte® Zoom software at the indicated time points. **J** SHH-MB-SLCs neurospheres were dissociated and electroporated with siRNAs to a non-relevant mRNA (siCTR) or murine *SALL4* (siSALL4). Representative bright field images of tumor neurospheres were acquired with the IncuCyte® zoom software. Scale bar: 400 µm. **K** The proliferative capability of SHH-MB-SLCs is expressed as neurospheres-size (μm^2^). **L** Protein expression levels of SALL4, GLI1, stemness, and tumorigenic markers are shown with **M** relative densitometric analysis. Representative immunoblotting of *n* = 3 biological replicas with similar results are shown in B, G, and L. Vinculin- or actin-normalized densitometric analysis in C, H, and M represent the mean of *n* = 3 independent experiments ± SD. Data in E and I are normalized to cell scans at time 0 and expressed as FC and represent the mean of *n* = 3 independent experiments ± SD. Data in F are normalized to endogenous *Gapdh* and *Hprt* control expressed as FC respect to the control sample value and represent the mean of *n* = 3 independent experiments ± SD. *p < 0.05; **p < 0.01; ***p < 0.001; ****p < 0.0001 versus siCTR or shCTR calculated using two-sided Student’s *t*-test. Schematic representations in A and D have been created by BioRender.com.

We examined SALL4 protein levels in Gfap-cre/*Ptc*^fl/fl^ and Math1-cre/*Ptc*^fl/fl^ mouse models in which homozygous deletion of *Ptc* is restricted to neuronal stem cells (NSCs) or granule neural progenitors (GNPs), respectively (Fig. 6A) [40–44]. Interestingly, we found high expression of SALL4 in murine MB tissues derived from both conditional SHH-dependent mouse models when compared to the one in cerebella of healthy siblings (Fig. 6B, C, Supplementary Fig. S3A,B).

We assessed the effects of SALL4 silencing on the growth of primary SHH-MB cells freshly isolated from MBs spontaneously developed in Math1-cre/*Ptc*^fl/fl^ mice (Fig. 6D). Lentiviral-induced genetic depletion of *SALL4* significantly suppresses tumor cell proliferation (Fig. 6E) and correlates with the impairment of SHH signaling activity (Fig. 6F–H). In addition, we observed an increase in AcGLI1 levels, thus sustaining the cooperation between SALL4 and HDAC1 in the modulation of GLI1 acetylation (Fig. 6G, H). To further investigate this aspect, we combined *SALL4* genetic depletion with HDAC1 pharmacological inhibition by using MGCD0103 (a well-known HDAC1/2 inhibitor [45]) in primary SHH-MB cells. As shown in Fig. 6I, while *SALL4* genetic depletion or MGCD0103 treatment alone restrains tumor cell proliferation, their combination does not further induce the inhibition of SHH-MB cells proliferative capability. This observation indicates that both SALL4 and HDAC1 functions converge on the same regulatory mechanism that culminates in GLI1 protein deacetylation to trigger SHH pathway activation and promote tumor growth. Further, our data suggest that SALL4 plays a specific role in SHH-malignant proliferation. Indeed, although we found a weak expression of SALL4 in GNPs (the cells of origin of SHH-MB) at an early post-natal stage (P5) that is SHH-dependent, its genetic inhibition in P5-old GNPs does not impair physiological SHH-driven cell growth (Supplementary Fig. S3C-F).

Given the crucial role of SALL4 in stemness, we assessed if its modulation could affect stemness and clonogenic properties of SHH-MBs. To this end, we first cultured tumor cells from spontaneous MB of Math1-cre/*Ptc*^fl/fl^ mice as neurospheres (MB Stem-Like Cells, MB-SLCs) in EGF- and bFGF-free cultured medium to retain the characteristic of in vivo SHH-MB [46, 47]. Then, the genetic silencing of SALL4 was assessed by electroporating MB-SLC neurospheres with siRNA targeting murine SALL4 (or a control non-targeting siRNA). Interestingly, the clonogenic self-renewal ability of SHH-MB-SLCs decreases in *SALL4*-depleted neurospheres (Fig. 6J, K) as a consequence of reduced expression of GLI1, stemness (OCT4 and SOX2) and oncogenic (N-MYC) markers (Fig. 6L, M). Overall, these findings support the oncogenic properties of SALL4 in SHH-MB.

### SALL4 knock-down inhibits SHH-MB growth in vivo

**Fig. 7:**
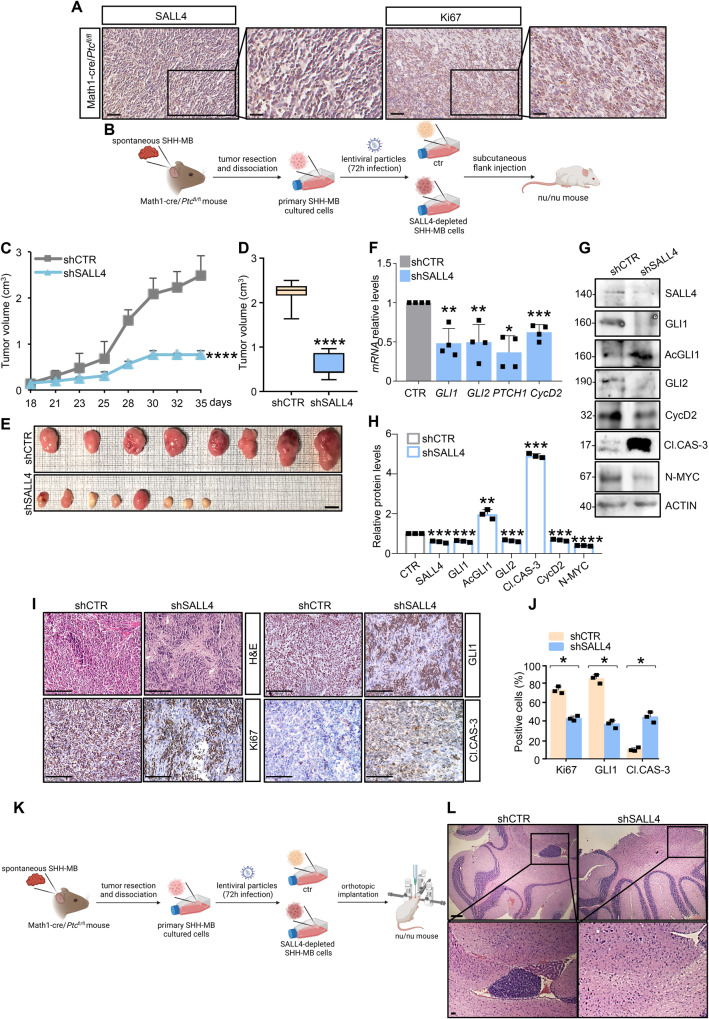
SALL4 inhibition impairs SHH-dependent tumor cell growth in vivo. **A** Representative images of SHH-MB tumors from Math1-cre/*Ptc*^fl/fl^ mice immune-stained with SALL4 or Ki67 used as control of proliferating cells (magnification ×40 and ×80; scale bars: 50 µm and 25 µm, respectively). **B** Primary SHH-MB cells have been infected with lentiviral particles expressing shSALL4 or shCTR. After infection, 2 × 10^6^ cells have been subcutaneously injected in both back flanks of nu/nu mice (*n* = 4/group). **C** Caliper measurements have been collected three times a week up to 35 days after injection to assess tumor growth. Quantification of **D** tumor explants and **E** representative flank allograft tumors are shown (magnification ×1; scale bar: 1 cm). **F** Relative transcript and **G** protein levels of SHH targets in explanted tumors are shown. **H** Protein levels are normalized to endogenous actin. **I** Representative hematoxylin and eosin (H&E) images and immunohistochemical staining of Ki67, GLI1, and cleaved Caspase-3 (CL. CAS-3) of representative tumor masses (magnification 20 ×; scale bar: 100 μm). **J** The Ki67, GLI1, and CL. CAS-3%-positive estimates have been calculated on the total of cells for each image. **K** Primary SHH-MB cells have been infected with lentiviral particles expressing shSALL4 or shCTR. After infection, 2 × 10^4^ cells have been orthotopically injected in the cerebellum of nu/nu mice (*n* = 6/group). **L** Representative H&E images (low and high magnifications) of murine SHH-MB orthotopic tumors derived from primary SHH-MB cells genetically silenced for SALL4 before the injection in nu/nu mice cerebella. (magnification ×4 and ×10; scale bars: 500 µm upper panel, and 200 μm lower panel). Representative immunoblotting of at least three independent tumor grafts with similar results are shown in G. Actin-normalized densitometric analysis in H represents the mean of *n* = 3 independent explanted tumors ± SD. Data in F are normalized to endogenous *Gapdh* and *Hprt* control expressed as FC respect to the control sample value and represent the mean of *n* = 4 tumor grafts ± SD. *p < 0.05; **p < 0.01; ***p < 0.001; ****p < 0.0001 versus shCTR calculated by two-sided Student’s t-test. Schematic representations in B and K have been created by BioRender.com.

Based on the evidence in cultured cells, we investigated the oncogenic role of SALL4 in vivo. As expected, immunohistochemistry analysis (IHC) reveals widespread expression of SALL4 in the high-proliferative area (positive to Ki67 staining) of SHH-MB tissues from Math1-cre/*Ptc*^fl/fl^ mice (Fig. 7A). We assessed tumor growth in heterotopic allograft animal model by subcutaneously injecting primary SHH-MB cells in both flanks of athymic nude mice (nu/nu) (Fig. 7B). Before injection, cells have been silenced for *SALL4* expression by lentiviral transduction. Grafts from control group develop progressively enlarging tumors, whereas SALL4-depleted tumor masses grow at a significantly slower rate (Fig. 7C–E). This effect strongly correlates with the reduction of SHH target genes and the increase of AcGLI1 levels (Fig. 7F–H). Consistently, tumor masses from the SALL4-silenced group show a reduced cellularity, a significant decrease in GLI1 and the proliferation marker Ki67 expression as well as increased apoptosis as indicated by increased expression of cleaved Caspase-3 (Cl. CAS-3) when compared to the control group (Fig. 7I, J). The pro-oncogenic properties of SALL4 have been also investigated in an orthotopic allograft model of SHH-MB. Primary SHH-MB cells have been implanted into the cerebella of nu/nu mice after lentiviral-mediated depletion of *SALL4* expression (Fig. 7K). As observed in Fig. 7L, whereas cells infected with the lentiviral particles expressing a non-targeting sequence (shCTR) give rise to detectable tumor masses, cells in which SALL4 has been silenced do not grow. These in vivo data confirm the tumorigenic role of SALL4 in the regulation of SHH-dependent tumor growth.

### SALL4 depletion represses human SHH-MB growth

**Fig. 8:**
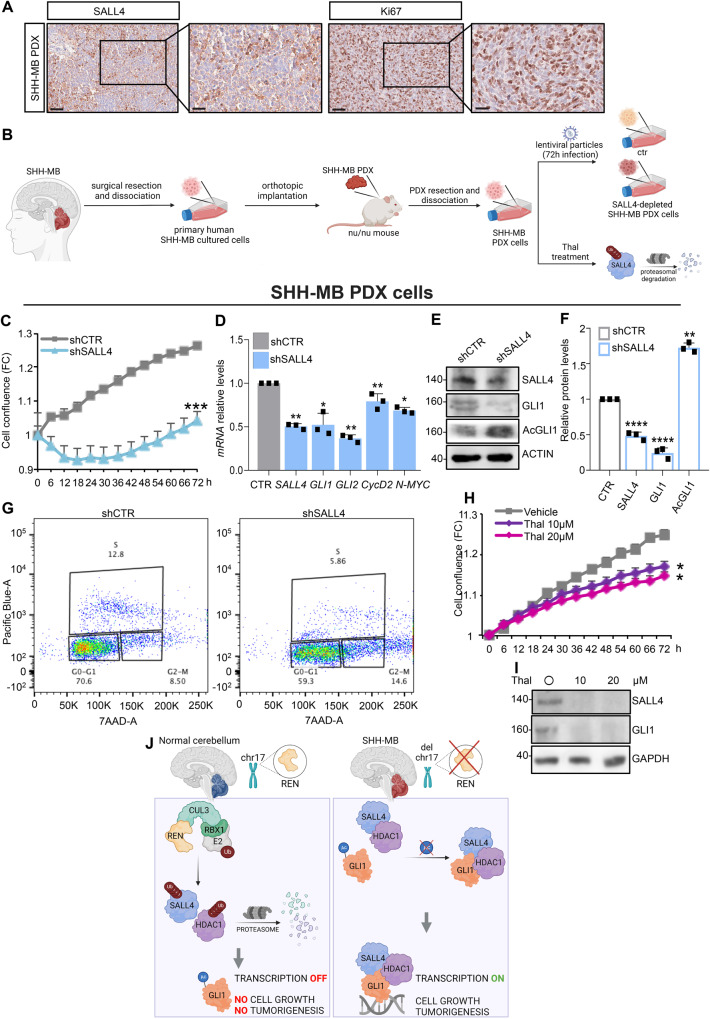
SALL4 inhibition counteracts growth of human MBs in SHH-MB PDX cells. **A** Representative images of SHH-MB PDX tumor samples immuno-stained with SALL4 and Ki67 used as control of proliferating cells (magnification ×40 and ×80; scale bars: 50 µm and 25 µm, respectively). **B** SHH-MB PDX cells have been infected with lentiviral particles (shCTR or shSALL4). **C** After infection, cell proliferation has been measured as cell confluence calculated with the IncuCyte® Zoom software by phase-contrast images. Cells proliferation is normalized to scans at time 0 and is expressed as FC. **D** mRNA expression of SHH signature and **E** immunoblotting of SHH related proteins, with **F** relative densitometric analysis are shown. **G** Cell cycle analysis by FACS of SALL4-depleted SHH-MB PDX cells. Plot data of cells labeled with Click-iT EdU Pacific Blue (y-axis) and Click-iT Cell Cycle 488-Red (7-ADD) (x-axis) fluorescence. Cells were analyzed by flow cytometer with 405 nm 450/50 nm bandpass and 675/20 nm bandpass, respectively. The percentage of cells in S phase, G_1_ phase, and G_2_ phase of cell cycle, are shown. **H** SHH-MB PDX cells have been treated with Thal (or vehicle) at the indicated concentrations. After treatment, cells proliferation has been measured as cell confluence calculated with the IncuCyte® Zoom software by phase-contrast images. Cells growth is normalized to scans at time 0 and is expressed as FC. **I** Immunoblotting of SALL4 and GLI1 levels are shown. Representative immunoblotting of *n* = 3 independent biological replicas with similar results are shown in E and I. Actin-normalized densitometric analysis in F represents the mean of *n* = 3 independent explanted tumors ± SD. Data in D are normalized to endogenous *Gapdh* and *Hprt* control expressed as the FC respect to the control sample value and represent the mean of *n* = 3 biological replicas ± SD. *p < 0.05; **p < 0.01; ***p < 0.001; ****p < 0.0001 versus shCTR calculated with two-sided Student’s t-test. **J** A representative model showing the new identified role of SALL4 in SHH-dependent tumorigenesis. In physiological condition, CRL3^REN^ promotes the ubiquitylation and proteasomal degradation of both SALL4 and HDAC1. This event maintains GLI1 acetylated thus impairing its transcriptional activity. As consequence, cells proliferation is blocked. In MB, the absence of REN allows SALL4 and HDAC1 protein accumulation. SALL4, HDAC1, and GLI1 are assembled in a trimeric complex, thus promoting GLI1 deacetylation and its transcriptional activity. This event triggers the SHH pathway activation and favors cell growth and tumorigenesis. Schematic representations in B and J have been created by BioRender.com.

Next, we validated the effects of SALL4 inhibition in human SHH-MB patient-derived xenograft (PDX) model. Investigating the intratumoral distribution of SALL4 in two independent SHH-MB PDXs, we found that SALL4 is detectable in the analysed tumor tissues (Fig. 8A, Supplementary Fig. S4A). Genetic depletion of *SALL4* in SHH-MB PDX cells (Fig. 8B) impairs cell proliferation compared to control (Fig. 8C), an effect associated to a reduction of SHH signature and an increase of AcGLI1 (Fig. 8D–F). The analysis of the cell cycle by Fluorescence-activated cell sorting (FACS) confirms that SALL4 depletion results in more than 3-fold decrease in the S phase compared to control cells (4.03% and 12.75%, respectively), underlying an impairment in DNA replication (Fig. 8G). At the same time, G2 population significantly increases in SALL4 depleted cells (13.75% versus 6.185%, in control cells) suggesting cell cycle arrest in G2/M phase (Fig. 8G).

Recent studies reported that SALL4 is a neo-substrate of thalidomide (Thal) [48], an immunomodulatory imide drug (IMiD) currently used in clinical practice for haematological and solid tumors. Thal efficiently directs SALL4 to Cullin4/Cereblon (CRL4^CRBN^)-mediated degradation [49, 50] exclusively in humans, primates, and rabbits [48]. Of interest, we found that Thal treatment impairs SHH-MB PDX cells proliferation (Fig. 8H), leading to SALL4 and GLI1 downregulation (Fig. 8I). Overall, these data confirm the relevance of SALL4 in SHH-MB growth and tumor prognosis.

## Discussion

The SHH signaling pathway is crucial for mammalian brain homeostasis and its aberrant activation is responsible for neurodevelopmental disorders and MB formation. The transcription factor GLI1 is the final effector of this signaling and regulates the transcriptional response to SHH. In addition, GLI1 can be activated by different oncogenic pathways thus highlighting the relevance to unveil the molecular mechanisms that govern its misregulation in cancer.

Ubiquitylation processes are crucial events by which GLI1 activity is finely regulated [38, 51–60]. Of note, we previously reported that the CRL3^REN^ E3 ligase complex finely suppresses GLI1 functions by promoting ubiquitylation and degradation of HDAC1, a strong activator of the SHH pathway [6].

The complexity of these regulatory mechanisms is a critical issue for the understanding of physiological SHH signaling activation and SHH-dependent tumorigenesis, thus prompting to identify and characterize novel players as targets for promising unexplored therapeutic options.

In this work we have identified SALL4, a master regulator of stemness and a well-recognized oncofetal protein, as a critical player of the SHH pathway. SALL4 is a zinc finger transcription factor mainly expressed in embryonic stem cells and implicated in the maintenance of pluripotency by its interaction with NANOG and OCT4, two of the main embryonic stemness markers [13, 14]. In mouse, loss of *SALL4* gene leads to embryonic lethality during implantation [61] and heterozygous *SALL4* mutant mouse recapitulate human Okihiro syndrome, an autosomal dominant disease with multiple developmental defects [62, 63]. Similar to mice, the expression of SALL4 is downregulated during development and rarely detectable in human adult tissues, but when reactivated it is a leading cause of a wide spectrum of cancers. An aberrant SALL4 activity has been reported in acute myeloid leukemia, lung adenocarcinoma, breast cancer, and other aggressive malignancies, and its overexpression is associated with poor prognosis and lower survival rate of patients [64–67]. Recently, it has been reported that knockdown of SALL4 in human melanoma cells decreases cell proliferation and impairs the expression of genes related to cell cycle, inflammation, and developmental processes [22]. For all these reasons, SALL4 is emerging as attractive therapeutic target in cancer.

Herein, we identified SALL4 as a novel substrate of REN, a CRL3 adaptor involved in the differentiation of GNPs, the cells of origin of SHH-MB [8, 10]. *REN* maps on chromosome 17p, a region frequently deleted in SHH-MB subgroup, and acts as tumor suppressor which, by promoting degradation of HDAC1, inhibits GLI1 activity and represses SHH-MB growth.

We demonstrated that, under physiological condition, REN binds SALL4 and HDAC1 and induces their ubiquitylation and degradation. This event results in the acetylation of GLI1 thus abrogating its function and suppressing cell growth. In SHH-MB, the loss of REN caused by chromosome 17p deletion, allows SALL4 and HDAC1 accumulation. Biochemical data demonstrate that SALL4, HDAC1, and GLI1 form a trimeric complex, thus promoting GLI1 deacetylation. This confers increased activity to GLI1, thereby enhancing SHH signaling and sustaining cell proliferation and tumor onset (Fig. 8J).

The relationship between SALL4 and HDACs in the regulation of gene expression represents a nodal point in tumor biology as well as an opportunity for cancer treatments. Pharmacological peptides that specifically disrupt the interaction between SALL4 and HDAC1 have been tested with success as therapeutic agents both in acute myeloid leukemia and hepatocarcinoma [65, 68]. Lung cancer cell lines expressing high levels of SALL4 are sensitive to the HDAC1 inhibitor Entinostat suggesting the use of this drug as a potential treatment for lung cancer [68]. Interestingly mocetinostat, a selective inhibitor of HDAC1 and HDAC2, drastically reduces SHH-MB growth in mouse models, an effect linked to GLI1 acetylation [45], thereby suggesting the potential of mocetinostat to counteract, at the same time, SALL4 activity.

Our work contributes to further understanding SALL4 functions and proposes alternative routes of intervention for SHH-MB, a highly heterogenous and aggressive malignancy of the cerebellum with a few treatment options. Despite the deep molecular characterization, the current therapies are based on surgery, radio- and chemotherapy; patients treated with the FDA-approved vismodegib, an antagonist of the SMO receptor, showed rapid development of drug resistance and severe side effects [4, 69]. Because of the existence of alternative mechanisms of activation to SMO, targeting downstream SHH components is now considered a preferable option. GLI1 inhibitors and multitargeting approaches [70, 71], including HDAC inhibitors, could offer a valuable opportunity to fight SHH-MB.

In the light of our findings, innovative strategies may arise from the use of drugs triggering SALL4 degradation. The recent discovery that the small immunomodulatory drug thalidomide induces ubiquitylation and degradation of SALL4 by the CRL4^CRBN^ E3 ubiquitin ligase [50] has demonstrated that this transcription factor can be targeted for cancer therapy [48, 50]. Efficacy of thalidomide and its derivatives has been demonstrated in neuroblastoma and multiple myeloma cell lines [48]. In addition, they are now in clinical trial evaluation in brain tumors, including MB (NCT01356290 [72], NCT03257631). Although thalidomide can target several substrates, our findings suggest that the thalidomide-dependent degradation of SALL4 may represent one mechanism contributing to the anti-tumor effects of this drug in SHH-MB. Overall, our studies unveil SALL4 as a novel regulator of SHH signaling and promising therapeutic target in SHH-MB.

## Material and methods

### Purification of REN/KCTD11 interactors

HEK293T cells (purchased by the American Type Culture Condition, ATCC) cells were transfected with pcDNA3-Flag-HA-REN and treated with MG132 10 µM for 5 h. Cells were harvested and subsequently lysed in lysis buffer (50 mM Tris-HCl pH 7.5, 150 mM NaCl, 1 mM EDTA, 0.5% NP40, plus protease and phosphatase inhibitors). REN was immunopurified with anti-Flag agarose resin (Sigma-Aldrich, St. Louis, MO, USA). After washing, proteins were eluted by competition with Flag peptide (Sigma-Aldrich). The eluate was then subjected to a second immunopurification with anti-HA resin (12CA5 monoclonal antibody crosslinked to protein G Sepharose; Invitrogen, Waltham, MA, USA) prior to elution in Laemmli sample buffer. The final eluate was separated by SDS-PAGE, and proteins were visualized by Coomassie colloidal blue. Bands were sliced out from the gels and subjected to in-gel digestion. Gel pieces were then reduced, alkylated and digested according to a published protocol [73]. For mass spectrometric analysis, peptides recovered from in-gel digestion were separated with a C18 column and introduced by nano-electrospray into the LTQ Orbitrap XL (Thermo Fisher Scientific, Waltham, MA, USA) with a configuration as described [74]. Peak lists were generated from the MS/MS spectra using MaxQuant build 1.0.13.13 [75], and then searched against the IPI Human database (version 3.37, 69164 entries) using Mascot search engine (Matrix Science). Carbaminomethylation (+57 Da) was set as fixed modification and protein N-terminal acetylation and methionine oxidation as variable modifications. Peptide tolerance was set to 7 ppm and fragment ion tolerance was set to 0.5 Da, allowing 2 missed cleavages with trypsin enzyme. Finally, Scaffold 3.6.1 (Proteome Software Inc.) was used to validate MS/MS based peptide and protein identifications. Peptide identifications were accepted if their Mascot scores exceeded 20.

### Cells and primary cultures

HEK293T and Med1-MB cells [29, 45] were cultured in Dulbecco’s Modified Eagle Medium (DMEM, Sigma-Aldrich) supplemented with 10% fetal bovine serum (FBS; Merck, Darmstadt, Germany). Media contained 1% Penicillin–Streptomycin (Pen–Strep) and 1% l-Glutamine.

Primary SHH-MB cells were freshly isolated from Math1-cre/*Ptc*^fl/fl^ mice tumors as described in [76] and cultured in Neurobasal Media-A (Thermo Fisher Scientific) with B27 supplement minus vitamin A (Thermo Fisher Scientific), 1% Pen–Strep and 1% l-Glutamine. PCR detection kit (Applied Biological Materials, Richmond, BC, Canada) was routinely used to test Mycoplasma contamination in cell cultures.

Stable SHH-dependent MB cells were cultured as neurospheres in DMEM/F12 media (2% B27 minus vitamin A; 3% Glucose 10×; 0.2% Insulin 10 mg/ml; 1% Pen/Strep; 0.01% Heparin 2 mg/ml; 0.06% N-Acetyl-L Cysteine) as described in Bufalieri et al. [47]. Whenever necessary, neurosphere cultures were pelleted and dissociated by incubation with Accutase (Sigma-Aldrich) to obtain a single cell suspension.

Cerebellar cultures of GNPs were obtained from 5-days old (P5) CD-1 mice. Cerebellum tissues were aseptically removed and incubated in digestion buffer (Dulbecco’s PBS with 0.1% trypsin, 0.2% EDTA, and 10 µg/ml DNase) for 15 min at room temperature. Then, tissues were mechanically disrupted to obtain a single-cell suspension and cells were seeded (2.5 × 10^5^ cells/cm^2^) in Neurobasal Medium (Thermo Fisher Scientific) 5% FBS supplemented with B27, 1% Pen–Strep and 1% l-Glutamine.

Patient-derived xenograft (PDXs) ICN-MB-PDX12 was generated from primary human SHH-MB tumor of patient diagnosed at the Children’s Necker Hospital in Paris and transplanted into the subscapular fat pad of immunocompromised NOD/SCID mice [77]. The SHH-MB PDX Med-1712FH was generated by the Olson lab [78]. Human SHH-MB PDX cells were obtained as described in [79]. Human samples were obtained with informed consent of patients, and all experimental procedures were performed following guidelines from the Institutional Review Board at Necker Hospital, Paris, France. Once established, PDX models were maintained by serial propagations in nu/nu mice. For in vitro cultures, tumors were dissociated in Neurobasal Media containing 1 mg/ml DNaseI (Worthington Biochemicals, Lakewood, NJ, USA), 2.5 mg/ml Collagenase P and 2.5 mg/ml Collagenase/dispase (Roche, Basil, Switzerland), B27 supplement minus vitamin A (Thermo Fisher Scientific), and N2 supplement (Invitrogen). Then, cells were cultured in Neurobasal Media supplemented with B27 supplement minus vitamin A, 0.01% BSA solution, 1% 1000X N-Acetyl Cysteine, and 1% d^+^-Glucose solution 45% (Sigma-Aldrich), 1% Pen–Strep and 1% l-Glutamine.

### Transfections and lentiviral infections

DreamFect^TM^ Gold or DreamFect^TM^ Transfection Reagents (Oz Biosciences SAS, Marseille, France) were used in accordance with the manufacturer’s protocols. siRNAs transfection in Med1-MB cells was performed by using HiPerFect Transfection Reagent (QIAGEN, Hilden, Germany). siRNAs electroporation in SHH-MB-SLCs was performed by using Mouse Neural Stem Cell Nucleofector® Kit (VPG-1004, Lonza Bioscience, Basel, Switzerland) in accordance with the manufacturer’s protocol. Silencer RNAs (Negative Control, AM4637; siSALL4 #1, MSS246807; siSALL4 #2, MSS246808; siREN/KCTD11, 170461) were purchased by Thermo Fisher Scientific.

Lentiviral particles were generated in HEK293 cells by transiently transfecting the packaging plasmids pCMV-dR8.74 and VSV-G/pMD2 with pLKO.1 plasmids (shCTR SHC002 or shSALL4 #TRCN0000097824 for primary murine SHH-MB cells, and #TRCN0000021878 for SHH-MB PDX cells, Sigma-Aldrich) using calcium phosphate transfection method. Cells were infected with purified lentiviral particles for 72 h.

### Plasmids, antibodies, and treatments

pcDNA3.1 GFP-/Flag-GLI1, Flag-/HA-HDAC1 and Flag-/HA-REN expressing vectors were generated in our lab by standard cloning techniques and verified by sequencing. pCDNA3.1 Flag-ΔBTB-, or BTB-REN mutants were constructed by deleting amino acids 18–80 or 196–232 to WT-REN, respectively. The following plasmids were kindly provided by other labs: pcDNA3.1 Myc-Cul3 (M. Pagano, New York University School of Medicine, USA), 12 × Gli–RE TKO-Luc, P1A WT-Luc, and P1A Mut-Luc (R. Toftgård, Karolinska Institutet, Sweden), pcDNA3.1 Flag-Ub (I. Dikic, Institute of Biochemistry Goethe University, Germany), pcDNA3.1 HA-SALL4 (W. Dai, New York University Langone Medical Center, USA). Single residues (pcDNA3.1 Flag-K518R GLI1) were mutated by the Quickchange site-directed mutagenesis kit (Agilent Technologies, Santa Clara, CA, USA). The N-terminal tagged 3xHA-6xHis SALL4 plasmids were designed and purchased by VectorBuilder (Neu-Isenburg, Germany). GFP-SALL4 WT (vector ID: VB230530-1241dyq); GFP-SALL4 ΔZFC1 (deleted in amino acids 320-486, vector ID: VB230530-1248kpp); GFP-SALL4 ΔZFC2 (deleted in amino acids 551-662, vector ID: VB230530-1278bkc); GFP-SALL4 ΔZFC4 (deleted in amino acids 859-1028, vector ID: VB230530-1264awg); GFP-SALL4 ΔNuRD (deleted in amino acids 1-12, vector ID: VB230530-1247eau).

Mouse anti-Gli1 (L42B10, 1:500 for WB; 1:100 for IHC) and rabbit anti-cleaved Caspase-3 (Asp175 D3E9, 1:100 for IHC, 1:1000 for WB) were purchased by Cell Signaling Technology (Beverly, MA, USA). β-Actin HRP (sc-47778, 1:2000), mouse anti-HA-probe F-7 HRP (sc-7392 HRP, 1:1000), mouse anti-Myc 9E10 (sc- 40, 1:500), mouse anti-Sall4 G-3 (sc-166033, 2 μg), rabbit anti-Gli1 H-300 (sc-20687, 1:100), mouse anti-Cyclin D1 C-20 (sc-717, 1:500) were purchased by Santa Cruz Biotechnology (Santa Cruz, CA, USA). HRP-conjugated secondary antibodies were purchased by Bethyl Laboratories (Waltham, MA, USA). Anti-Flag M2 HRP (A8592, 1:1000), rabbit anti-HDAC1 (H3284, 1:1000), and rabbit anti-Flag (F7425, 2 μg) were purchased by Sigma-Aldrich. Goat anti-Gli2 (AF3635, 1:1000) was purchased by R&D Systems (Minneapolis, MN, USA). Rabbit anti-Sall4 (ab29112, 1:1000 for WB, 1:100 for IHC) was purchased by Abcam (Cambridge, UK). Rabbit anti-Ki67 SP6 (MA5-14520, 1:100) was purchased by Thermo Fisher Scientific. Rabbit anti-Acetyl-Gli1(Lys518) antisera (1:500 for WB) was generated by Eurogentec by rabbit immunization with the peptide acetylated-Gli1(Lys518) H2N-IGS RGL K(Ac)LPSLT CCONH2 [39]. The specificity of the antibody was validated by competition assay with the immunogenic peptide, with or without lysine acetylation. Anti-mouse Alexa Fluor 546 (A11003, 1:400) was purchased by Life Technologies (Foster City, CA, USA).

MG132 (Calbiochem, Nottingham, UK), cycloheximide (CHX, 100 μg/ml up to 8 h; Sigma-Aldrich), mocetinostat (MGCD0103, 0.5 μM up to 72 h; synthetized in house as a dihydrobromide salt as described in [80]), thalidomide (Thal, 10–20 μM up to 72 h; Tocris Bioscience, Bristol, UK), and Smoothened Agonist (SAG, 200 nM, Alexis Biochemicals, Farmingdale, NY, USA) were used were indicated.

### Luciferase reporter assays

In vitro functional transcription assays were performed in HEK293Ts transiently transfected with a *Firefly* luciferase reporter containing 12 binding sites for GLI1 in its synthetic promoter (12 × GLI1-BS-Luc) or *Ptc*-dependent luciferase reporter with a conserved or mutated GLI1 binding site in its promoter (P1A WT-Luc or P1A Mut-Luc), pRL-TK *Renilla* and indicated plasmids. 24 h after transfection, a dual-luciferase assay system was used to analyze the expression signals of Firefly and Renilla following the manufacturer’s instructions (Biotium Inc., Hayward, CA, USA). Results were expressed as Luciferase/Renilla ratios and represented the mean ± S.D. of at least *n* = 3 experiments, each performed in triplicate.

### Immunoblot analysis and immunoprecipitation

Protein lysates were obtained in RIPA buffer (50 mM Tris-HCl at pH 7.6, 150 mM NaCl, 0.5% sodium deoxycholic, 5 mM EDTA, 0.1% SDS, 100 mM NaF, 2 mM NaPPi, 1% NP-40) supplemented with protease and phosphatase inhibitors. The lysates were centrifuged at 13,000 rpm for 30 min at 4 °C and the resulting supernatants were boiled for 5 min in loading buffer. The protein extracts were then separated by SDS-PAGE, transferred to nitrocellulose membranes (GVS North America, Sanford, ME, USA), blocked with 5% skimmed milk in TBS containing 0.1% Tween 20 (Sigma-Aldrich), and incubated with the indicated antibodies.

For co-immunoprecipitations, cells were lysed as described above, quantified, and at least 1 mg of the whole-cell protein extracts was incubated overnight at 4 °C with specific primary antibodies or IgG used as a control (2 μg/mg; Santa Cruz Biotechnology). The day after, immunocomplexes were incubated with G- or A-Protein agarose beads (Santa Cruz Biotechnology) for 1 h at 4 °C. The IPs were then washed five times, and samples were prepared for SDS-PAGE resolving and then subjected to immunoblot analysis. Uncropped Western blots are provided in Supplementary Material.

### In vivo ubiquitylation assays

HEK293T cells were lysates with a denaturing buffer (1% SDS, 50 mM Tris-HCl at pH 7.5, 0.5 mM EDTA, 1 mM DTT). NETN buffer (100 mM NaCl, 20 mM Tris-Cl pH 8.0, 0.5 mM EDTA, 0.5% (v/v) NP-40) was used to dilute 10 times the lysates during immunoprecipitation (from 2 h to overnight at 4 °C) with indicated antibodies. To perform the immunoblot analysis, the IPs were washed with NETN buffer, resuspended in sample loading buffer, boiled for 5 min, resolved in SDS-PAGE, and then subjected to immunoblot analysis to detect the polyubiquitylated forms. Uncropped Western blots are provided in Supplementary Material.

### Cell proliferation assays

Med1-MB cells were transiently transfected for 24 h with si- or shRNAs where indicated, while primary murine SHH-MB cells and human SHH-MB PDXs were infected with lentiviral particles encoding either short hairpin RNA targeting SALL4 (shSALL4) or a control non-targeting sequence (shCTR) for 72 h. 1 × 10^3^ cells/well for Med1-MB cells, 2 × 10^4^ cells/well for primary murine MB cells, and 1 × 10^5^ SHH-MB PDX cells were seeded onto a 96-well tissue culture plate in 100 μl complete medium (6 wells for each experimental point). Med1-MB cell proliferation was measured as cell confluence (%), while primary SHH-MB cell proliferation was indicated as relative Nuclight staining (Nuclight Rapid Red reagent, #4717, Sartorius, Gottinga, Germany), both calculated using the IncuCyte® Zoom software (Essen BioScience Ann Arbor, MI, USA). Cells proliferation is normalized to scans obtained at time 0 (T0) and expressed ad fold change (FC) ± SD of *n* = 3 experiments. Med1-MB cells were scanned every 3 hours up to 72 h after transfection; both murine primary SHH-MB and SHH-MB PDX cells were scanned every 6 h up to 96 h and 72 h after infection, respectively.

Proliferation of SAG-induced GNPs was evaluated by Click-iT™ EdU Cell Proliferation Kit for Imaging (#C10337, Thermo Fisher Scientific) according to the manufacturer’s protocol.

### Flow cytometry and cell cycle analyses

Cell cycle analysis was performed using the Click-iT™ EdU Pacific Blue flow cytometry assay kit (Thermo Fisher Scientific) according to the manufacturer’s protocol. In brief, 10 μM of 5-ethynyl-2′-deoxyuridine (EdU) was added into culture medium, and SHH-MB PDX cells were incubated for 1 h at 37 °C. Then, cells were fixed with 4% paraformaldehyde for 60 min, and EdU was labeled with Pacific Blue. 7-Aminoactinomycin D (7-AAD) was added for measuring DNA content and cell cycle distribution. Data were collected on an LSR II or BD FACSVantage flow cytometer using FACSDiva software (both from BD Immunocytometry Systems) and analyzed using FlowJo software (Tree Star).

### mRNA expression analysis

Total RNA was isolated from cells using TRIzol reagent (Invitrogen). Synthesis of first-strand cDNA was performed by reverse transcription of total RNA using SensiFAST cDNA Synthesis Kit (Bioline, London, UK) according to manufacturer’s protocol. The ViiA^TM^ 7 Real-Time PCR System (Life Technologies Carlsbad, CA, USA) was employed to perform quantitative real-time PCR analysis (qRT-PCR) of the indicated mRNA expression levels. The reaction mix containing the cDNA template, the SensiFAST Probe or SYBR® Lo-ROX Kit (Bioline, London, UK) and the Taqman gene expression assays (Thermo Fisher Scientific) or the primer probes was amplified using standard qPCR thermal cycler parameters. Each sample was amplified in triplicate and the quantification of the mRNA was performed using SDS version 2.3 software. The average of the three threshold cycles was used to calculate the number of transcripts. Data were normalized with the endogenous housekeeping genes (*GAPDH* and *HPRT*) and expressed as the FC respect to the control sample value. The following qRT-PCR assays were used: *Sall4* (mSall4 forward, CCCCTCAACTGTCTCTCTGC; mSall4 reverse, CAGGGAGCTGTTTTCTCGA; hSALL4 forward, ATTTGTGGGACCCTCGACAT; hSALL4 reverse, TTAAGTTGCCTTTGGTGGTAA); *Gli1* (Mm00494654_m1; Hs00171790_m1; mGli1 forward, AAGCCAACTTTATGTCAGGG; mGli1 reverse, AGAGCCCGCTTCTTTCTTAA); *Gli2* (Mm01293117_m1; Hs01119974_m1); *Ptch1* (Mm00436026_m1; Hs0018117_m1); *Ccnd2* (Mm00438070_m1; Hs00153380_m1); *N-Myc* (Mm00476449_m1; Hs00232074_m1); *Ren/Kctd11* (Mm00628328_s1); *Hprt* (Mm00446966_m1; Hs02800695_m1; mHprt forward, GCTTCCTCCTCAGACCGCTT; mHprt reverse, GGTCATAACCTGGTTCATCATC); *Gapdh (*Mm99999915_g1; Hs02786624_g1).

### Immunohistochemistry

For IHC analysis, tissues were fixed and the slides were stained as reported in [47]. Briefly, tissues were first fixed in formalin and embedded in paraffin (FFPE) and then incubated overnight at 4 °C with anti-SALL4, anti-GLI1, -cleaved Caspase-3 or -Ki67 antibodies. The next day, the slides were incubated for 20 min with secondary antibodies coupled with peroxidase (Dako), which is then detected by the diaminobenzidine (DAB) solution (ScyTek Laboratories, Logan, UT, USA) and the EnVision FLEX Substrate buffer containing peroxide (Dako, Agilent, Santa Clara, CA, USA). Cell quantification was performed on stained sections with NIS-Elements BR 4.00.05 (Nikon Instruments Europe B.V., Florence, Italy) imaging software. Stained slides were scanned using the NanoZoomer S60 Digital slide scanner C13210-01 (Hamamatsu Photo- nics). Scanned images were viewed and captured with Hamamatsu Photonics’s image viewer software (NDP.view2 Viewing software U12388-01) at indicated magnifications.

### Animal studies

Female nu/nu mice of 28–34 days (086NU/NUCD1) were purchased by Charles River Laboratories (Calco, LC, Italy). CD-1, Gfap-Cre/*Ptc*^fl/fl^, and Math1-cre/*Ptc*^fl/fl^ mouse models, previously described by [41], were already available in our animal husbandry.

For in vivo heterotopic allograft experiments, spontaneous SHH-MB from Math1-cre/*Ptc*^fl/fl^ mice were disaggregated, and cells were cultured as described above. Then, primary cells were infected with purified lentiviral particles expressing shSALL4 or a scramble non-targeting sequence as control (shCTR). After infection, 2 × 10^6^ cells were subcutaneously injected (s.c.) on both posterior flanks of nu/nu mice randomly divided in two groups (*n* = 4). Cells were resuspended in an equal volume of culture medium and Matrigel® Basement Membrane Matrix (#354248, BD Biosciences, Heidelberg, Germany) before injection. Changes in tumor volume were evaluated with the formula (length × width) × 0.5 × (length + width), measured with caliper at indicated days.

For in vivo orthotopic allograft models, nu/nu mice were anesthetized by intraperitoneal (i.p.) injection of ketamine (10 mg/kg) and xylazine (100 mg/kg). The posterior cranial region was placed in a stereotaxic head frame and primary infected SHH-MB cells (shSALL4 or shCTR) were stereotaxically implanted into the cerebellum (2 × 10^5^/3 μl) according to the atlas of Franklin and Paxinos coordinates (*n* = 6 mice for each experimental group). After injection, at an infusion rate of 1 μl/min, the cannula was kept in place for 5 min. 45 days after tumor implantation animals were sacrificed and brains were fixed in 4% formaldehyde and paraffin embedded. Tumor volume calculation was performed on serial 40 coronal sections of 2 μm after hematoxylin and eosin (H&E) staining every 40 μm of brain slice. A microscope (Axio Imager M1 microscope; Leica Microsystems GmbH, Wetzlar, Germany) equipped with a motorized stage and Image Pro Plus 6.2 software was used to evaluate tumor area of each slide. All animal protocols were approved by local ethic authorities (Ministry of Health) and conducted in accordance with Italian Governing Law (D.lgs 26/2014). We followed the European and national regulations for the care and use of animals to protect them for experimental and other scientific purposes (D.lgs 26/2014).

### Datasets and data analyses

Through the R2 Genomics Analysis and Visualization Platform (http://r2.amc.nl), we analyzed the expression levels of KCTD11 and SALL4 in SHH-MB subgroup and SHH-MB alfa, beta, delta, and gamma subtypes using the previously generated dataset “Tumor Medulloblastoma – Cavalli – 763 – rma_sketch – hugene11t”, accession number: GSE85217. The survival distribution was estimated according to the Kaplan-Meier method and the significance was determined using log-rank statistics. The log-rank test was used for comparison of patient survival between high and low expression groups for each selected gene. Statistical significance was defined as *p* ≤ 0.05.

### Statistical analysis

Statistical analyses were performed with GraphPad Prism software version 9 (GraphPad, San Diego, CA, USA). *P*-values were determined by two-tailed Student’s *t*-test for all in vitro experiments. For animal studies, statistical significances were determined by two-way ANOVA and the sample size determination was accounted on the need for statistical power. Statistical significance was set at *p* ≤ 0.05. For IncuCyte® experiments, data were analyzed with the IncuCyte® software package (Essen BioScience, UK).

### Supplementary information

Supplementary Figures

Uncropped Western Blots

## Data Availability

All data in this study are available within the article and Supplementary Information or from the corresponding authors on reasonable request.
