# Supplementary material for: SALL4 is a CRL3REN/KCTD11 substrate that drives Sonic Hedgehog-dependent medulloblastoma
Source: Cell Death Differ. 2023 Dec 7;31(2):170–87. doi: 10.1038/s41418-023-01246-6 (PMC10850099; doi:10.1038/s41418-023-01246-6)
Supplement: Supplementary file 1 — Supplementary Figures [file 41418_2023_1246_MOESM1_ESM.pdf]

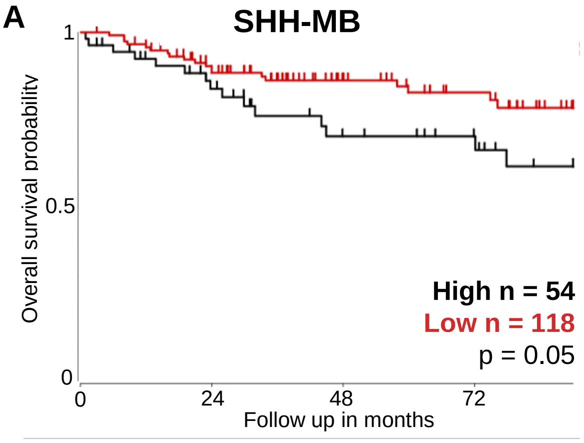

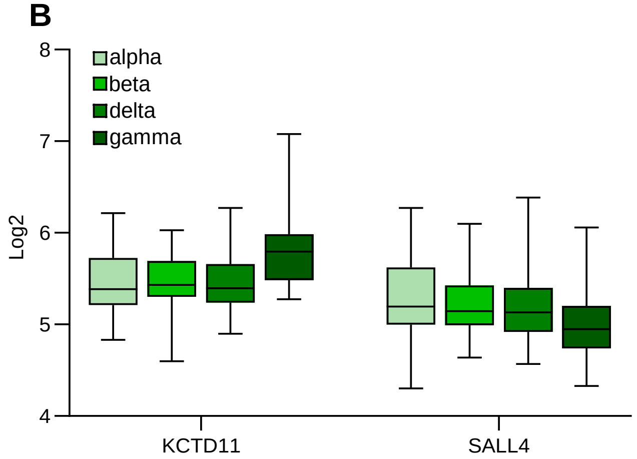

SHH-MB subtypes	KCTD11	SALL4
Alpha *vs* Beta	0,7846	0,8132
Alpha *vs* Delta	0,6392	0,5253
Alpha *vs* Gamma	**<0,0001**	**0,0006**
Beta *vs* Delta	0,9232	0,7438
Beta *vs* Gamma	**<0,0001**	**0,0028**
Delta *vs* Gamma	**<0,0001**	**0,0006**

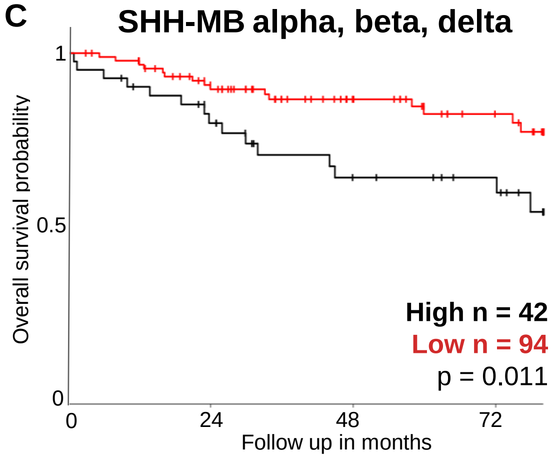

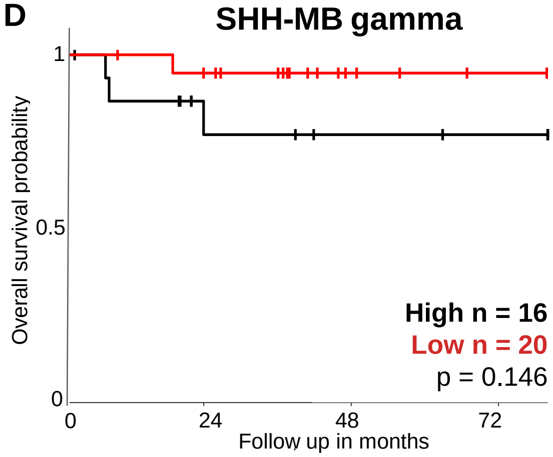

Fig. S1 Association between SALL4 expression and SHH-MB patients' overall survival. (**A,C,D**). Analysis of the overall SHH-MB patient survival (Kaplan-Meier curves) and *SALL4* expression levels using optimal cut-off selection and log- rank statistics. Data are from the R2: Genomics Analysis and Visualization Platform (http://r2.amc.nl), retrieved from the Cavalli database (accession number: GSE85217). (**B**) Box-dot plot showing mRNA expression levels of *REN/KCTD11* and *SALL4* (R2 platform) in SHH-MB subtypes (retrieved from Cavalli database); statistical analysis of are on the right. Data are represented as means ± SD and analyzed by two-way ANOVA.

A

Identified Protein	Accession Number	EV	REN
REN	Q693B1	0	20
CUL3	Q13618	0	22
RBX1	P62877	0	3
KCTD15	Q96SI1	0	2
SALL4	Q9UJQ4	0	1

B **C**

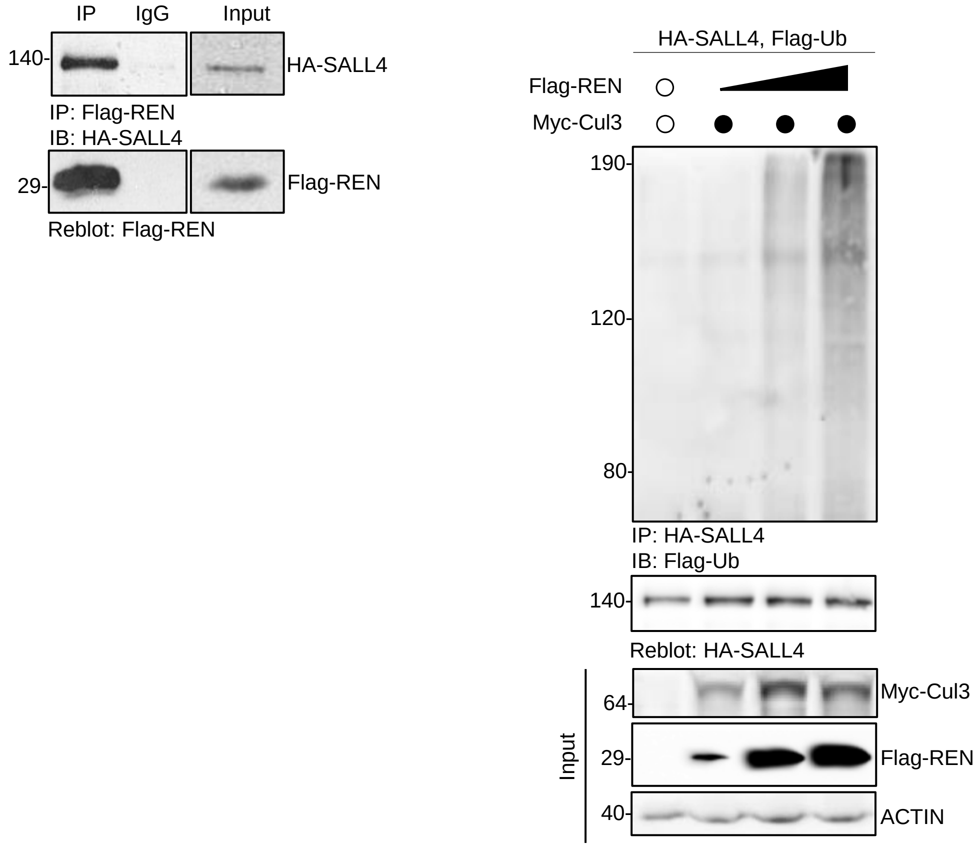

Fig. S2 REN binds and ubiquitylates SALL4. (**A**) FLAG-HA epitope tagged REN or an empty vector (EV) were expressed in HEK293T cells and immunoprecipitated. Immunocomplexes were then analyzed by mass spectrometry. The number of unique peptides identified by mass spectrometry in REN and control immunopurifications are shown. (**B**) REN interacts with SALL4. Co-IP of exogenous REN and SALL4 in HEK293Ts transiently transfected with indicated plasmids. (**C**) IP of HA-SALL4 from HEK293Ts transiently transfected with indicated plasmids. anti-Flag antibody was used to detect the SALL4 polyubiquitylated forms; anti-HA antibody was used to re-probe blot to assess the levels of immunoprecipitated protein. Total protein lysates are shown in the Input. Representative blots of n = 3 biological replicas with similar results are shown.

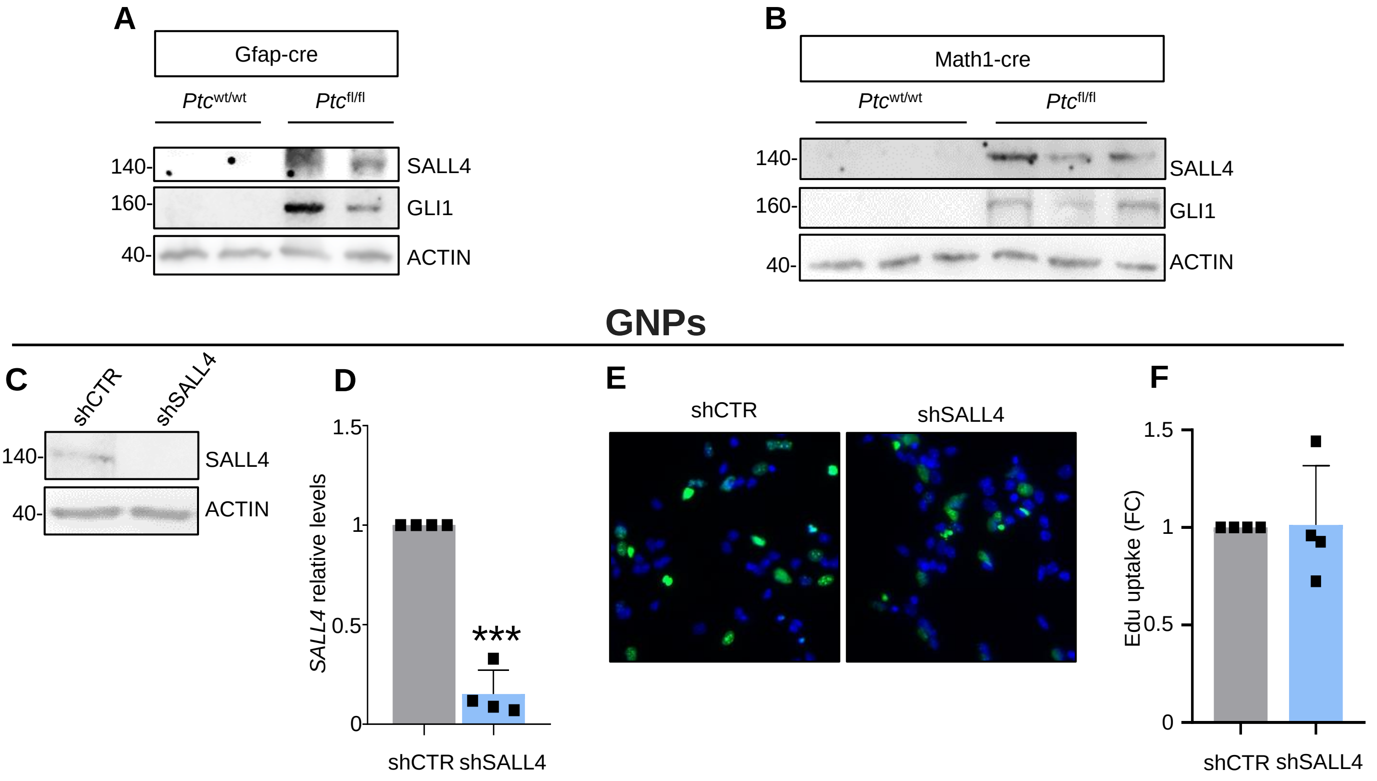

Fig. S3 Expression of SALL4 in mouse SHH-MBs. SALL4 levels have been evaluated in protein lysates of SHH-MB tissues from Gfap-cre/*Ptc*$^{fl/fl}$ (n = 2, **A**) and Math1-cre/*Ptc*$^{fl/fl}$ mice (n = 3, **B**) compared to the cerebella of healthy siblings (n = 2, **A**; n = 3, **B**). **SALL4 does not affect SHH-dependent growth of cerebellar granule cell progenitors.** (**C,D**) GNPs were isolated from 5-day-old mice, treated with the SMO-agonist SAG and infected with lentiviral particles encoding for shSALL4 or the corresponding control (shCTR). (**E**) Representative images of Edu incorporation (magnification 40×; scale bar: 50 μm) and (**F**) Edu uptake (expressed as FC) are shown. Representative immunoblotting of n = 4 biological replicas with similar results are shown. Data in D are normalized to endogenous *Gapdh* and *Hprt* control expressed as the FC respect to the control sample value. *** $p < 0.001$ calculated with two-sided Student's t-test.

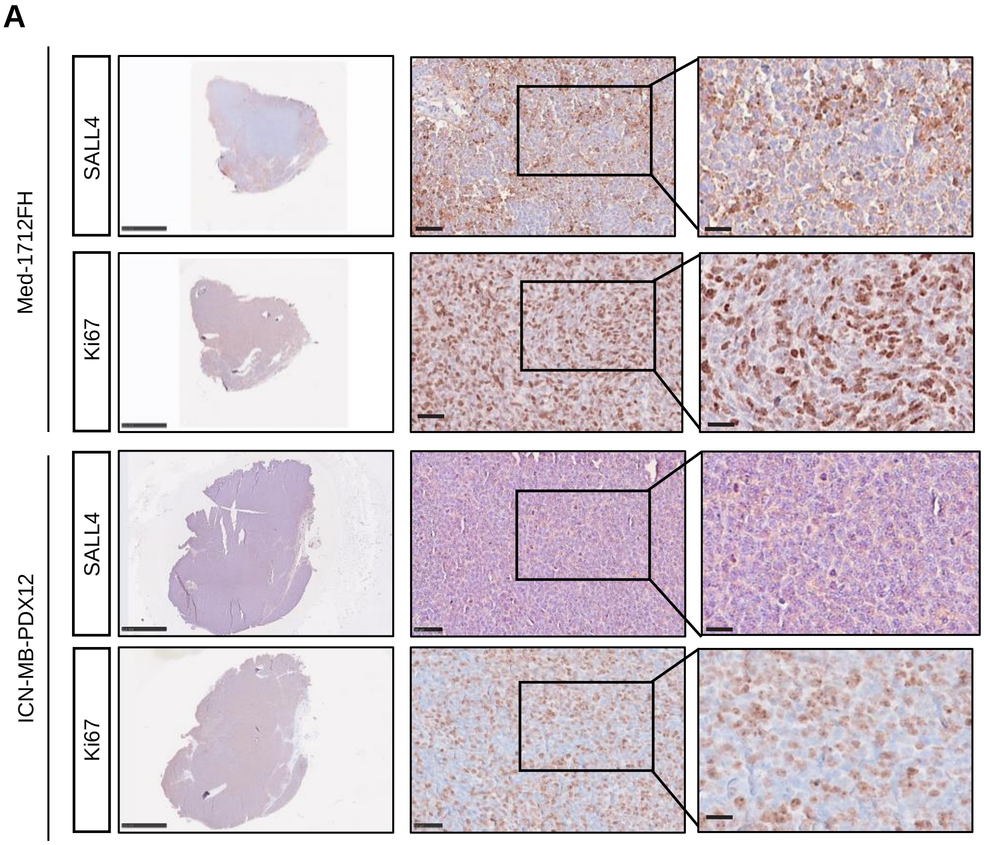

Fig. S4 Expression of SALL4 in human SHH-MBs. (**A**) Representative images of SHH-MB PDX tumors (ICN-MB-PDX12 and Med-1024 1712FH) immunostained with SALL4 or Ki67 (magnifications 1 x, 40 x, and 80 x; scale bars: 2 500 µm, 50 µm, and 25 µm, respectively).
