# Supplementary material for: SALL4 is a CRL3REN/KCTD11 substrate that drives Sonic Hedgehog-dependent medulloblastoma
Source: Cell Death Differ. 2023 Dec 7;31(2):170–87. doi: 10.1038/s41418-023-01246-6 (PMC10850099; doi:10.1038/s41418-023-01246-6)

Fig. 1B

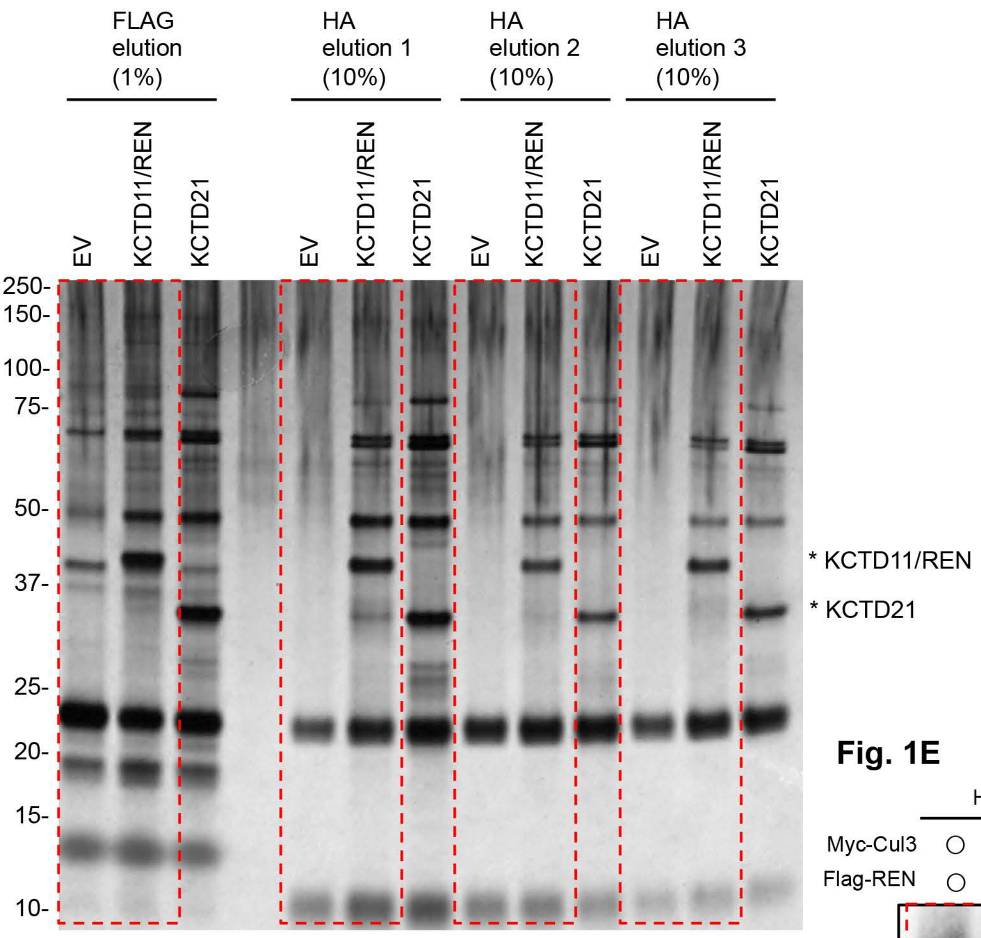

Fig.1C

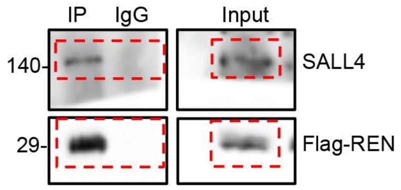

Fig.1D

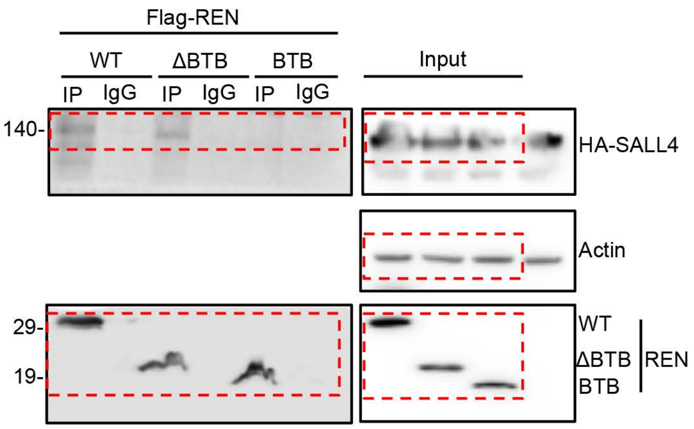

Fig. 1E

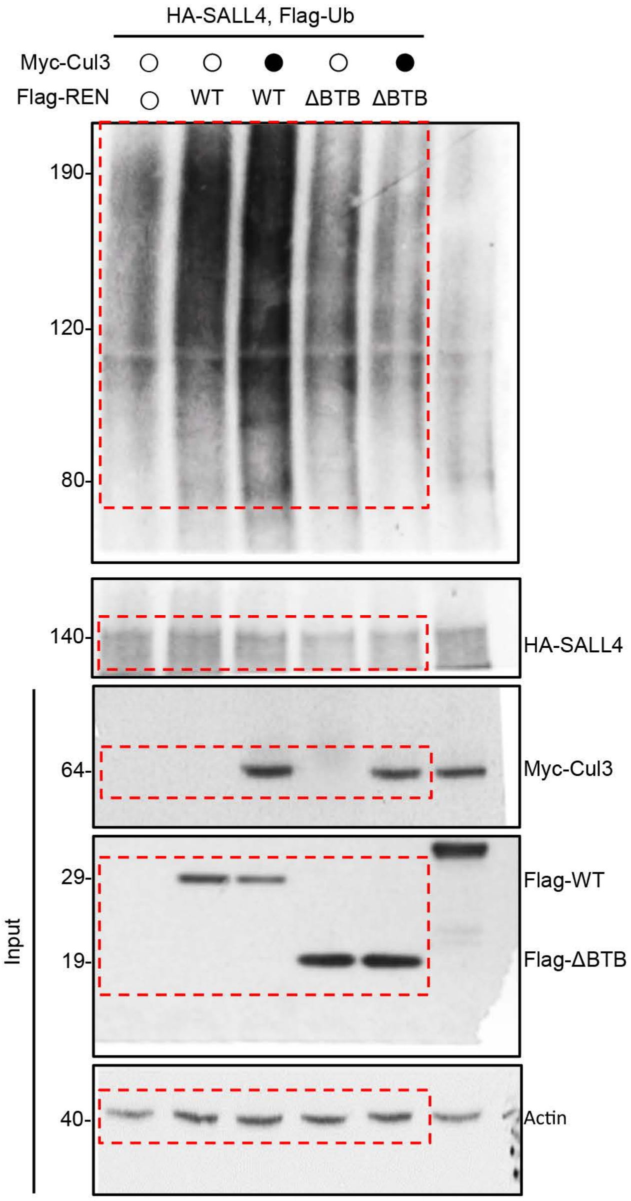

Fig. 1F

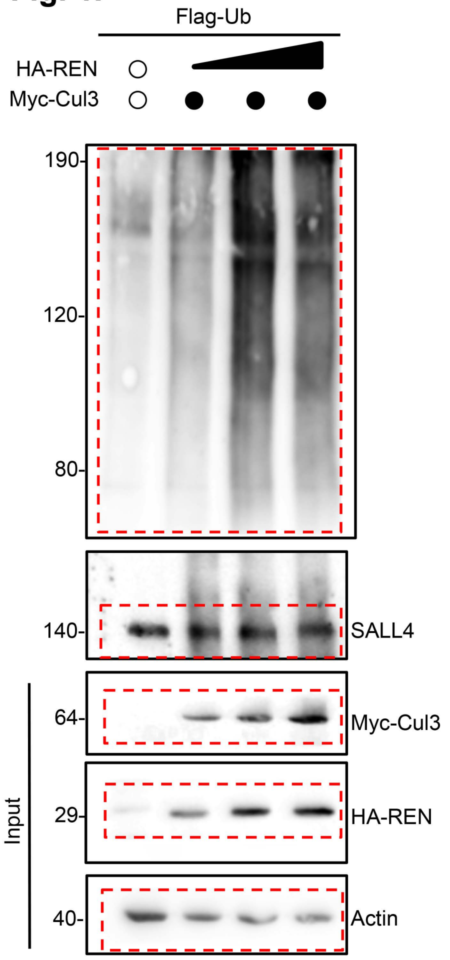

Fig. 1G

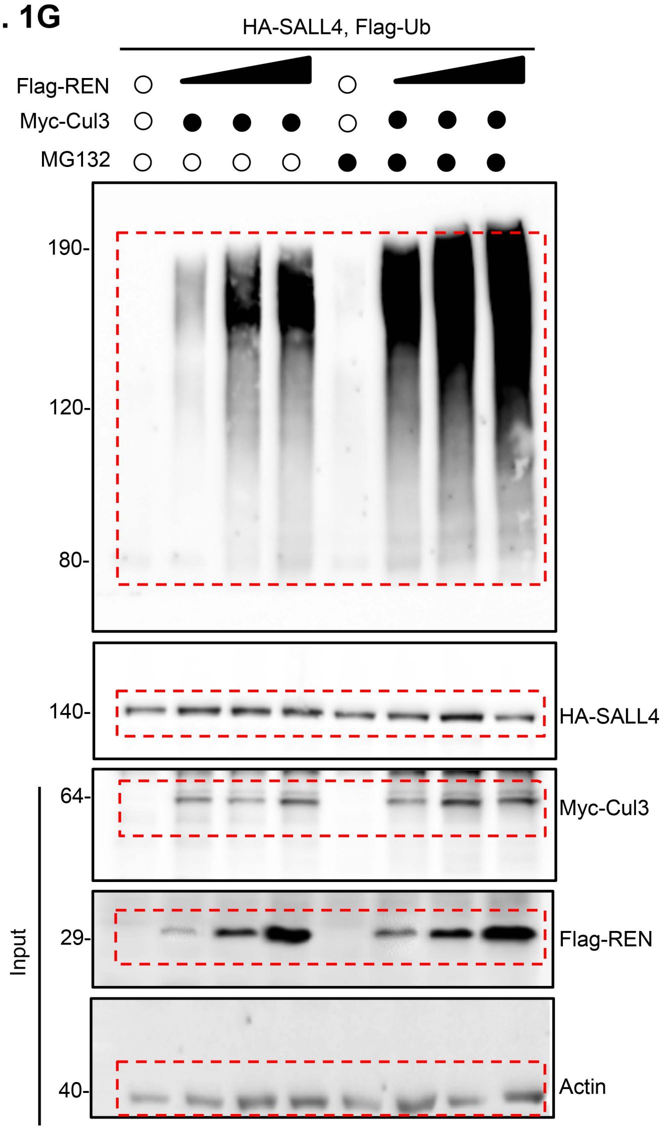

Fig. 1H

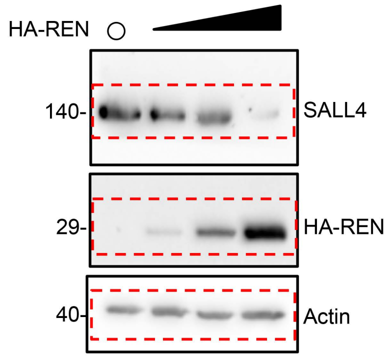

Fig. 1K

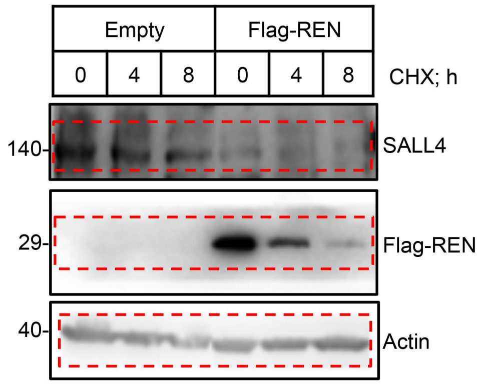

Fig. 2B

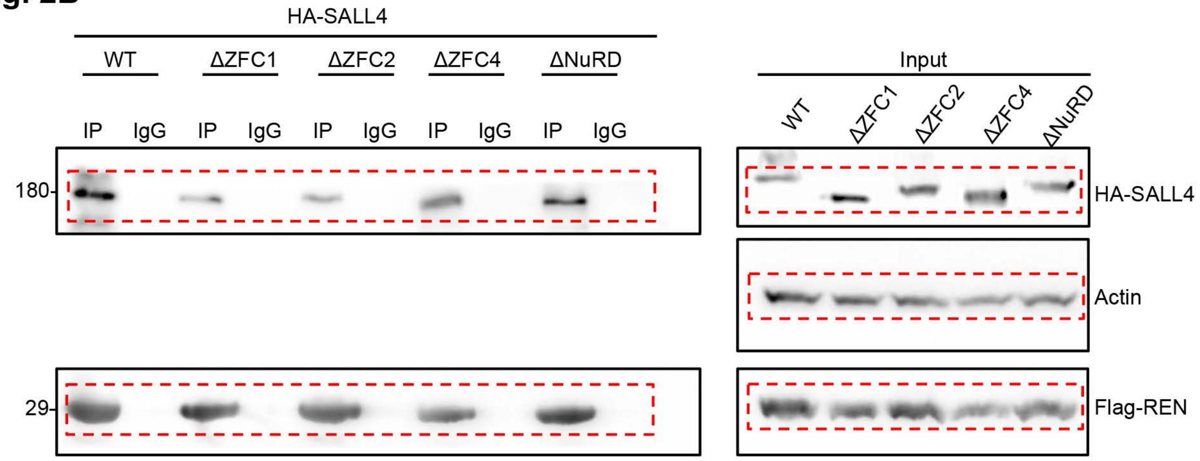

Fig. 2D

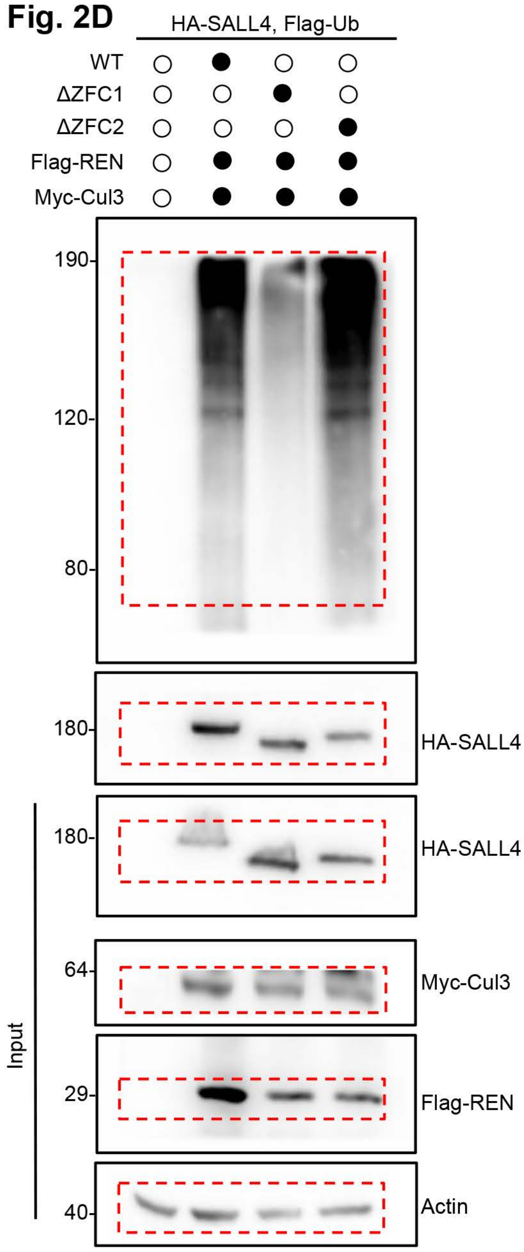

Fig. 2E

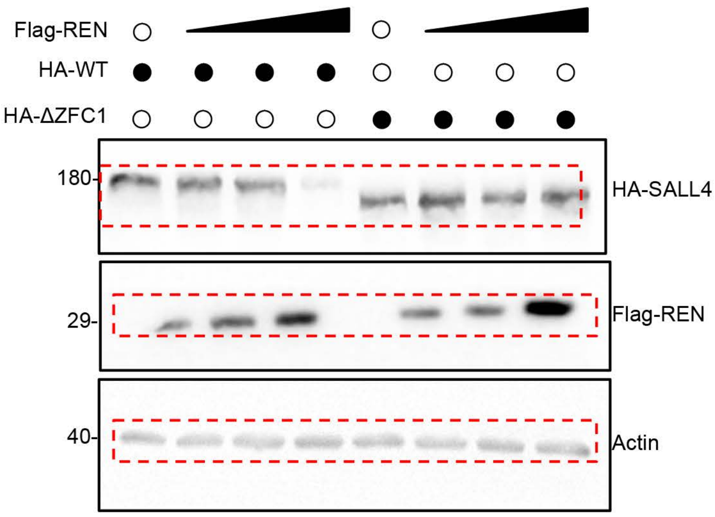

Fig. 2G

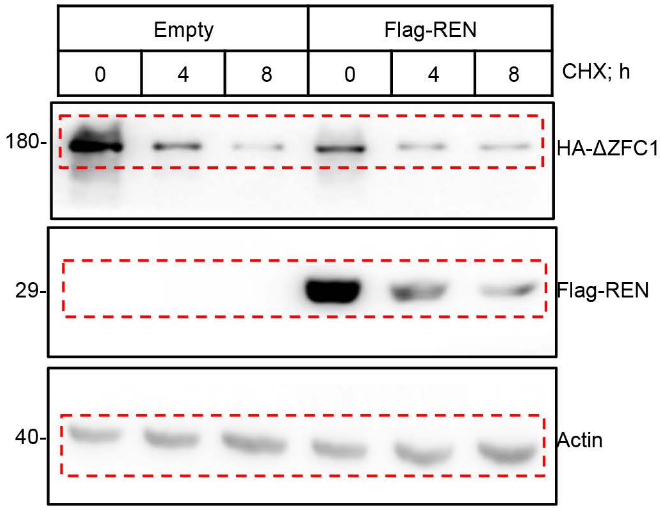

Fig. 3A

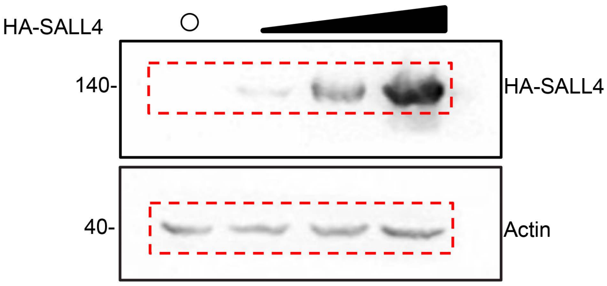

Fig. 3B

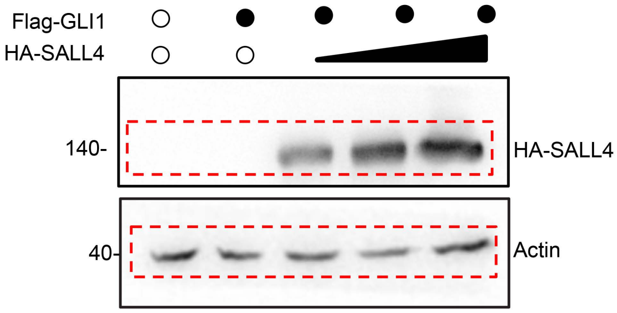

Fig. 3C

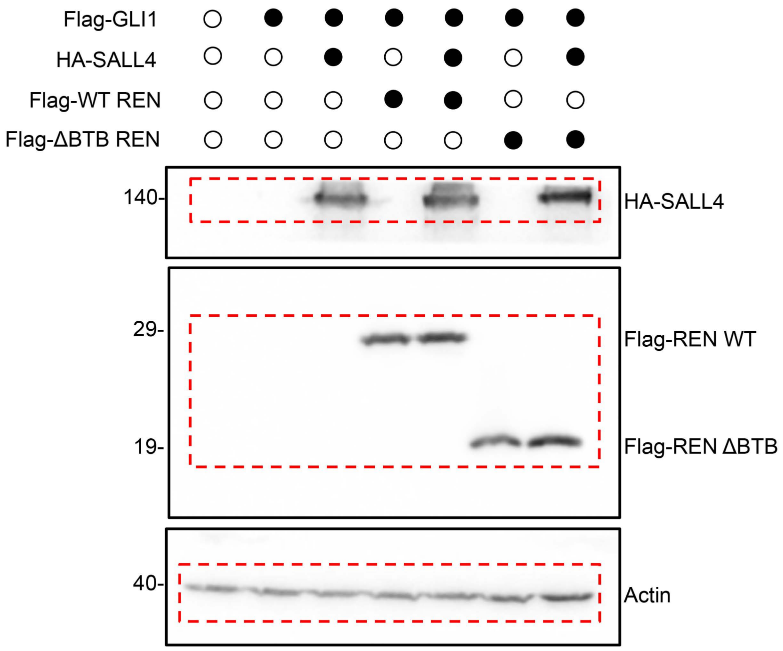

Fig. 3D

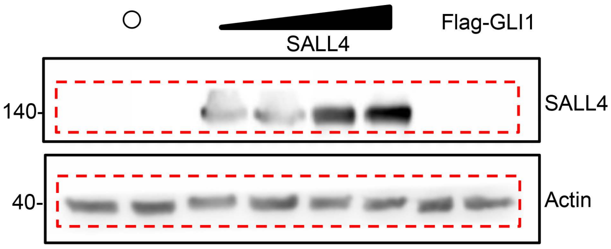

Fig. 4B

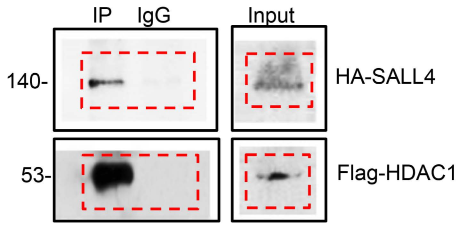

Fig. 4C

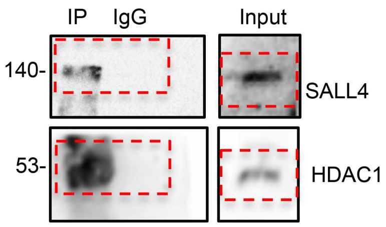

Fig. 4D

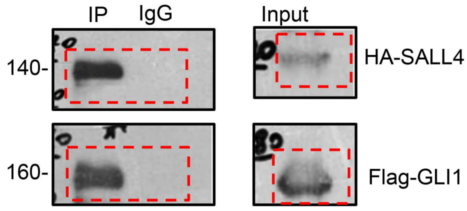

Fig. 4E

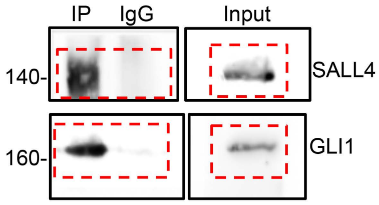

Fig. 4F

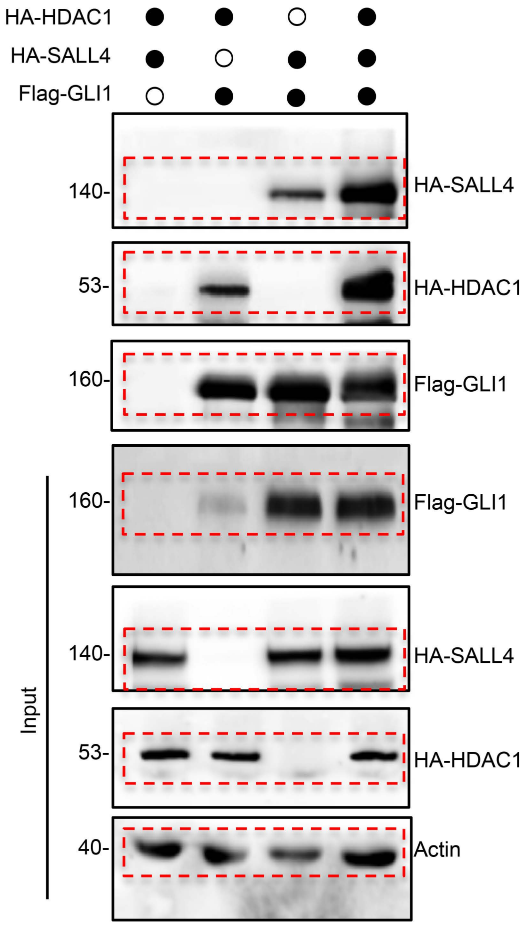

Fig. 4H

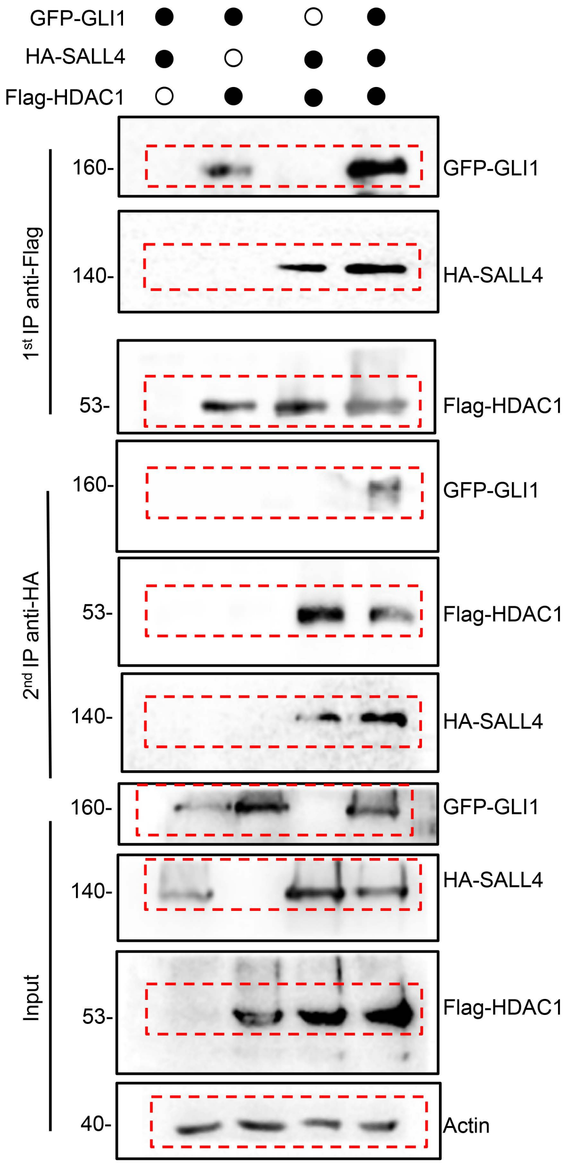

Fig. 4I

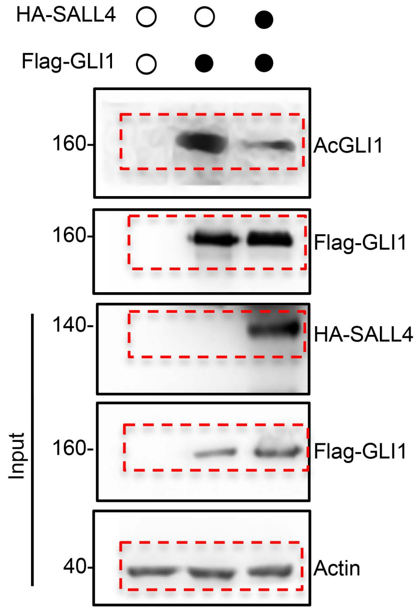

Fig. 5C

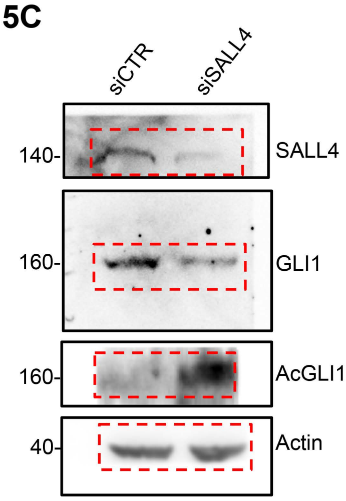

Fig. 5G

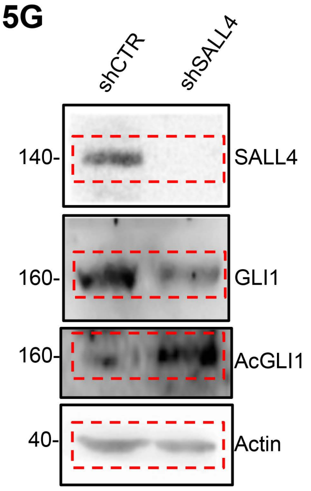

Fig. 5J

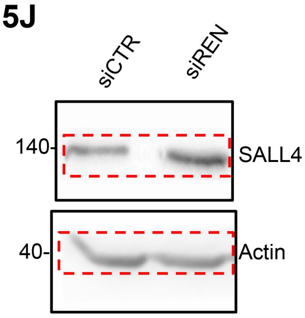

Fig. 5M

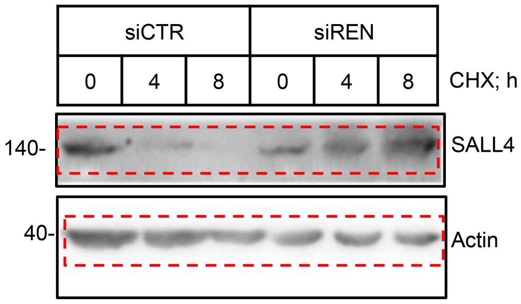

Fig. 6B

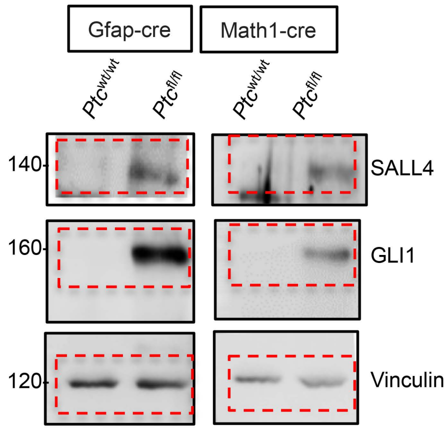

Fig. 6G

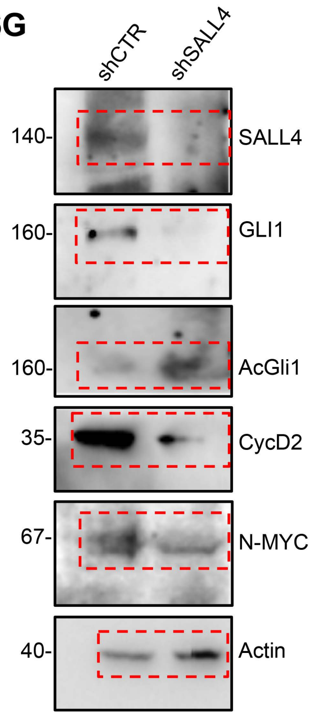

Fig. 6L

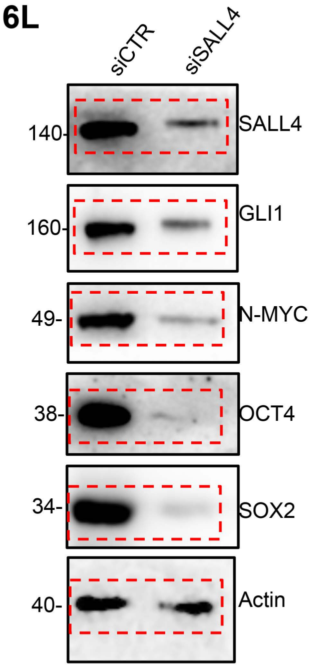

FIG. 7G

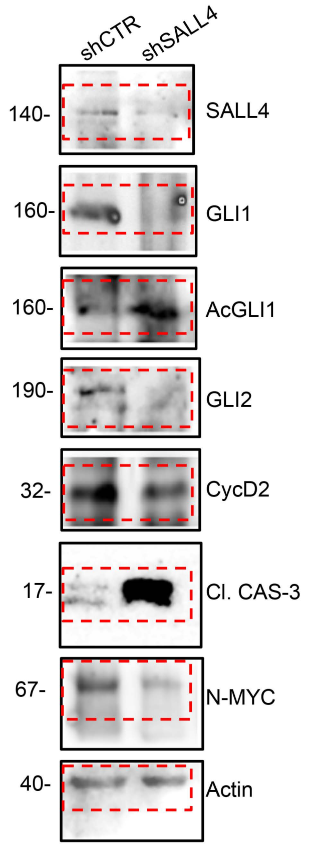

FIG. 8E

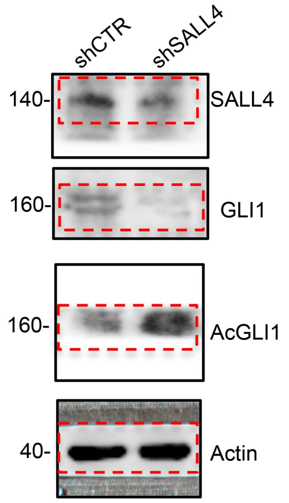

FIG. 8I

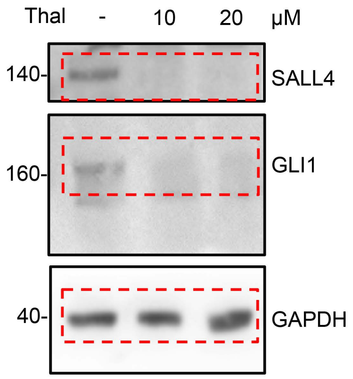

Fig. S2B

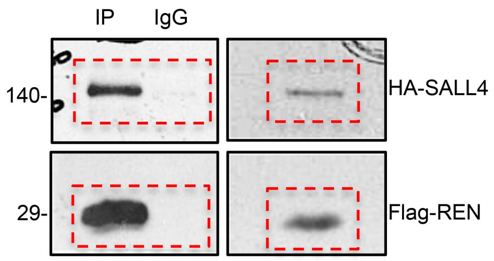

Fig. S3C

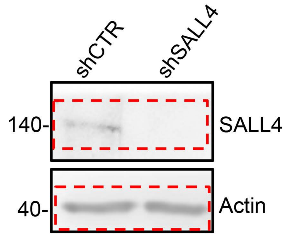

Fig. S2C

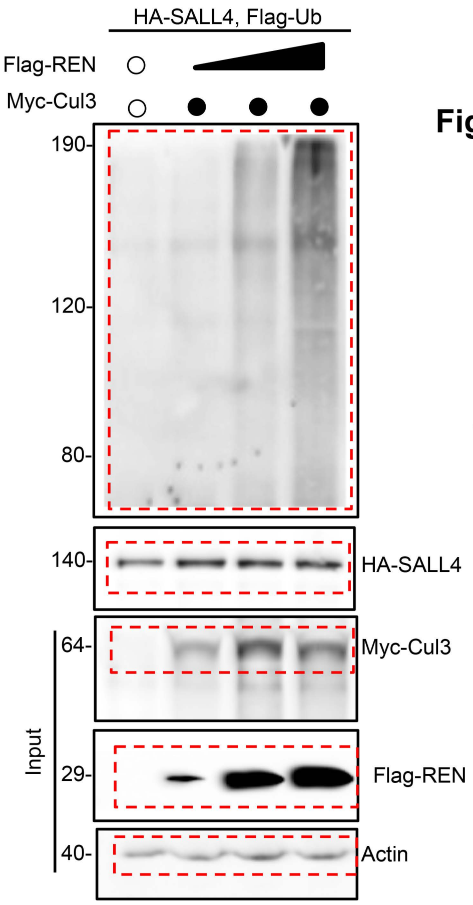

Fig. S3A

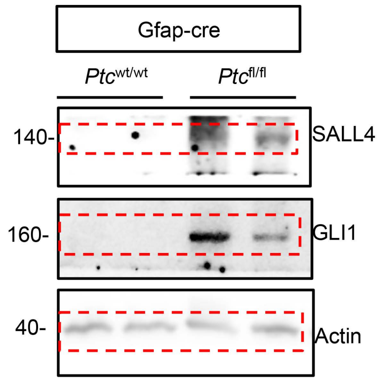

Fig. S3B

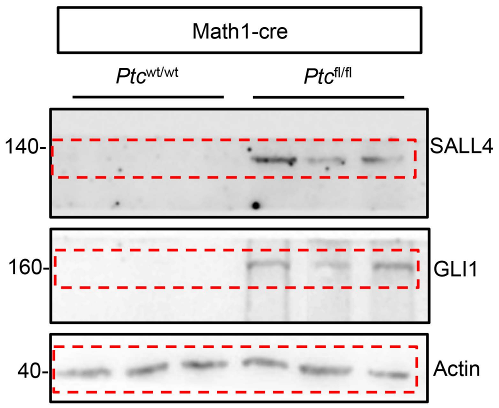

Supplement: Supplementary file 2 — Uncropped Western Blots [file 41418_2023_1246_MOESM2_ESM.pdf]
